# Role of (p)ppGpp in Viability and Biofilm Formation of *Actinobacillus pleuropneumoniae* S8

**DOI:** 10.1371/journal.pone.0141501

**Published:** 2015-10-28

**Authors:** Gang Li, Fang Xie, Yanhe Zhang, Janine T. Bossé, Paul R. Langford, Chunlai Wang

**Affiliations:** 1 Division of Bacterial Diseases, State Key Laboratory of Veterinary Biotechnology, Harbin Veterinary Research Institute, Chinese Academy of Agricultural Sciences, Harbin, China; 2 Section of Paediatrics, Department of Medicine, Imperial College London, St. Mary’s Campus, London, United Kingdom; University Medical Center Utrecht, NETHERLANDS

## Abstract

*Actinobacillus pleuropneumoniae* is a Gram-negative bacterium and the cause of porcine pleuropneumonia. When the bacterium encounters nutritional starvation, the *relA*-dependent (p)ppGpp-mediated stringent response is activated. The modified nucleotides guanosine 5’-diphosphate 3’-diphosphate (ppGpp) and guanosine 5’-triphosphate 3’-diphosphate (pppGpp) are known to be signaling molecules in other prokaryotes. Here, to investigate the role of (p)ppGpp in *A*. *pleuropneumoniae*, we created a mutant *A*. *pleuropneumoniae* strain, S8*ΔrelA*, which lacks the (p)ppGpp-synthesizing enzyme RelA, and investigated its phenotype *in vitro*. S8*ΔrelA* did not survive after stationary phase (starvation condition) and grew exclusively as non-extended cells. Compared to the wild-type (WT) strain, the S8*ΔrelA* mutant had an increased ability to form a biofilm. Transcriptional profiles of early stationary phase cultures revealed that a total of 405 bacterial genes were differentially expressed (including 380 up-regulated and 25 down-regulated genes) in S8*ΔrelA* as compared with the WT strain. Most of the up-regulated genes are involved in ribosomal structure and biogenesis, amino acid transport and metabolism, translation cell wall/membrane/envelope biogenesis. The data indicate that (p)ppGpp coordinates the growth, viability, morphology, biofilm formation and metabolic ability of *A*. *pleuropneumoniae* in starvation conditions. Furthermore, S8Δ*relA* could not use certain sugars nor produce urease which has been associated with the virulence of *A*. *pleuropneumoniae*, suggesting that (p)ppGpp may directly or indirectly affect the pathogenesis of *A*. *pleuropneumoniae* during the infection process. In summary, (p)ppGpp signaling represents an essential component of the regulatory network governing stress adaptation and virulence in *A*. *pleuropneumoniae*.

## Introduction

*Actinobacillus pleuropneumoniae* is a non-motile Gram-negative bacterium causing porcine pleuropneumonia, a highly contagious respiratory disease that is transmitted through aerosols or close contact with infected animals including asymptomatic carriers. This disease is often fatal and characterized by hemorrhagic, fibrinous and necrotic lung lesions; the clinical features ranging from acute to chronic, and it is an important cause of economic losses worldwide in the porcine industry [1].

The stringent response is a broadly conserved bacterial stress response that controls adaptation to nutrient deprivation, and is activated by a number of different starvation and stress signals. This response is used by bacteria to determine resource allocation for either reproductive or cell maintenance functions [2]. It is important for activation of survival strategies such as the stationary phase, sporulation and biofilm formation [3–5]. The central molecular signals of this response are the small molecules guanosine 5’-diphosphate 3’-diphosphate (ppGpp) and guanosine 5’-triphosphate 3’-diphosphate (pppGpp) (together termed (p)ppGpp) [6, 7]. To regulate the concentration of (p)ppGpp, some bacteria express RelA, which phosphorylates GDP or GTP to produce (p)ppGpp, or hydrolyzes (p)ppGpp back to GDP or GTP, to allow growth after nutrient restrictions are alleviated [7].

The stringent response is also utilized by many bacterial pathogens to regulate their virulence. Recently, a growing number of studies identified the stringent response as being important for both virulence and survival in harsh environments [8–11]. The complexity and multiplicity of the bacterial genes and regulatory pathways affected by the stringent response suggest that the relationship between the stringent response and virulence could be considerably more complex than expected and is perhaps unique for each pathogen [12].

*A*. *pleuropneumoniae* can adhere to cells of the lower respiratory tract in a process involving different adhesins and probably biofilm formation [13]. In this site, *A*. *pleuropneumoniae* causes tissue damage leading to clinical disease and mortality [13]. After successful adherence, *A*. *pleuropneumoniae* requires a variety of nutrients to sustain growth and exert its pathogenic effects. However, the lower respiratory tract is a nutrient-limited environment [14]. Subashchandrabose *et al*. [15] previously reported that most amino acids were present in lower concentrations in epithelial lining fluid compared to serum, and some amino acids (lysine and threonine) were present only at roughly 40 to 50% of the serum level.

Transition between growth in the upper respiratory tract and lung tissue subjects *A*. *pleuropneumoniae* to environmental stresses [16]. However, it is poorly understood how *A*. *pleuropneumoniae* can withstand such stresses. In particular, it is not yet known whether the stringent response has a role in stress adaption and/or is necessary for virulence traits of *A*. *pleuropneumoniae* within the porcine respiratory tract. In the present study, we have inactivated the *relA* gene (required for (p)ppGpp synthesis) in *A*. *pleuropneumoniae* strain S8 [17], and compared its growth, morphology, metabolic and enzyme activity, viability, ability to form biofilms, and transcriptome with its wild-type parent. The results suggest that (p)ppGpp directly or indirectly affects the pathogenesis of *A*. *pleuropneumoniae*.

## Materials and Methods

### Bacterial strains, primers, plasmids and growth conditions

The bacterial strains, primers and plasmids used in this study are described in Table 1. All *A*. *pleuropneumoniae* strains were cultured in Tryptic Soy Broth (TSB) or Tryptic Soy Agar (TSA) (Becton Dickinson, Franklin Lakes, NJ, USA) supplemented with 10 μg /ml NAD [18]. Selection of *A*. *pleuropneumoniae ΔrelA* transformants was achieved by the addition of chloramphenicol (5 μg/ml) to TSA. Complemented *A*. *pleuropneumoniae* S8*ΔrelA* HB was grown in a TSB supplemented with NAD (10 μg/ml), chloramphenicol (5 μg/ml) and kanamycin (50 μg/ml). For culture of *E*. *coli* β2155 (*ΔdapA*), Luria-Bertani (LB) medium was supplemented with 1 mM diaminopimelic acid (DAP) (Sigma-Aldrich, St. Louis, MO, USA) and, when required, chloramphenicol (30 μg/ml). All the bacteria were cultured at 37°C.

**Table 1:** Strains, plasmids and primers used in this study.

Strains	Description	Reference
S-8	*A*. *pleuropneumoniae* serovar 7 clinical isolate from the lung of a diseased pig in northern China	[17]
S-8*ΔrelA*	Inactivation of S8 (*relA*) by insertion mutant	This study
S-8HB	The complemented strain of *A*. *pleuropneumoniae* S8*ΔrelA* containing the *relA* gene	This study
*E*. *coli* β2155	*thrB1004 pro thi hsdS lacZ M15* (F’ *lacZ M15 lacI* ^*q*^ *traD36 proA* ^+^ *proB* ^+^)*dap*::*erm* (Erm^r^)	[19]
**Plasmids**		
pEMOC2	Transconjugation vector based on pBluescript SK with *mobRP4*, a polycloning site, Cm^r^, and transcriptional fusion of the *omlA* promoter with the *sacB* gene	[20]
pLS88	Broad-host-range shuttle vector from *Haemophilus ducreyi*; Str^r^ Sm^r^ Km^r^	[21]
pEMOC2-*ΔrelA*	900 bp homologous fragment of *relA* cloned into pEMOC2 via SalI*/*NotI sites	This study
pLS*relA*	pLS88 with a PCR-derived insert containing the *relA* gene	This study
**Primers**		
P1	5′GTCGACATAAGTAAAGCCGAAGCGAGCCATC3′, Mutagenesis	This study
P2	5′GCGGCCGCTGGAACGCGAGAGTTATATCGCAAA3′, Mutagenesis	This study
P3	5′ATACCGCTTCGTCTTCTTGTTCTGC3′, Confirmation of mutation, comprising bases 1684–1708 of the *relA* gene	This study
P4	5′CTTCAACCCAACACCGGACAAAAAG3′, Confirmation of mutation, comprising bases 2803–2827 of the pEMOC2 plasmid	This study
P5	5′GAATTCGACCGCTTGTCACTTAATTTGATAA3′, Complement, 13 bp downstream of the stop codon of the *relA* gene	This study
P6	5′GAGCTCGCAAATAATTGTAGAGATTGGTTTG3′, Complement, 200 bp upstream of the start codon of the *relA* gene	This study
*gyrA-F*	5′CAAGCGAATGCAGCTGTTTA3′	[22]
*gyrA-R*	5′CTGTGATGCCGTAGAGGACA3′	[22]
*recF-F*	5′TATGCCGAGATTCTTGCTCA3′	[22]
*recF-R*	5′AATTTAAGCTGCCCACGAGA3′	[22]
*relA-F*	5′TGCAAATGTCACCGATAACCTTT3′	This study
*relA-R*	5′GAGTCATCATCCATATTCAGCCC3′	This study

### Construction of a (p)ppGpp synthase deletion mutant and complemented strain

**Fig 1:**
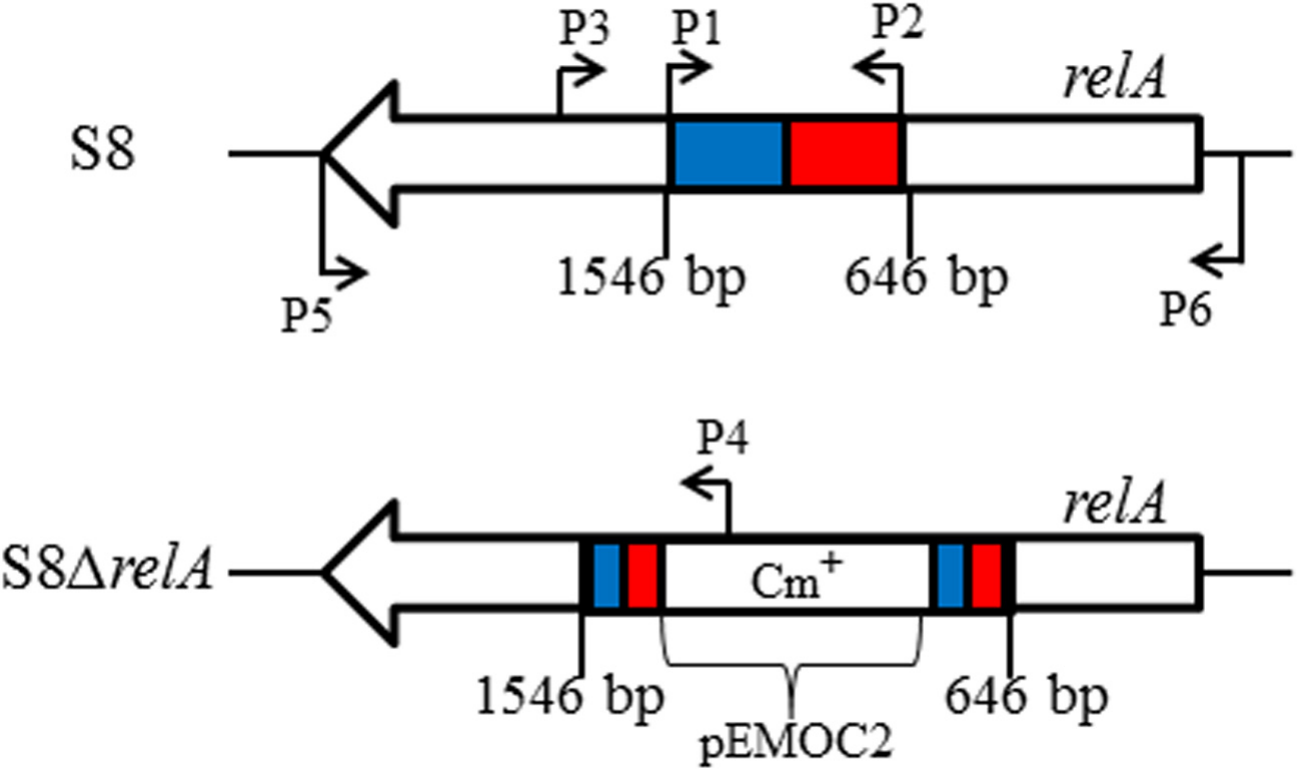
Schematic representation of the construction of S8*ΔrelA* mutant. The figure shows the binding locations for the oligonucleotide primers used to amplify the homologous regions (from 646 bp to 1556 bp) used in the construction of the insertion plasmid. The blue area represents the upstream region of the homologous fragment; the red area represents the downstream region of the homologous fragment.

The strategy used for inactivation of the *relA* gene in *A*. *pleuropneumoniae* was as described previously [23]. A 900-bp DNA fragment of *relA* (646 bp-1546 bp, encoding amino acid residues 216 to 516 of the RelA protein) was amplified from genomic DNA of *A*. *pleuropneumoniae* strain S8 with primers P1 and P2 (Table 1, Fig 1). The PCR product was cloned into the suicide plasmid pEMOC2 between sites SalI and NotI. The resulting insertional plasmid, pEMOC2-*ΔrelA*, was electroporated into *A*. *pleuropneumoniae* S8. Recombinants were selected on TSA plates containing chloramphenicol (5 μg/ml). The *ΔrelA* strain was verified to have the plasmid inserted into the *relA* locus by PCR using primers P3 and P4 and DNA sequencing of the resulting amplicon. To construct the complemented strain, full-length *relA* gene with its signal peptide sequence was amplified from S8 genomic DNA with primers P5 and P6 (Table 1, Fig 1). The PCR product was cloned between the EcoRI and the SacI sites of the shuttle vector pLS88. The recombinant plasmid, pLS*relA*, was confirmed by DNA sequencing and electroporated into the S8*ΔrelA* strain. Transformants were selected on TSA plates containing kanamycin (50 μg/ml) and chloramphenicol (5 μg/ml). The complemented strain was confirmed by PCR and DNA sequencing of the amplicon, and named S8HB.

### Detection of intracellular (p)ppGpp

The production of (p)ppGpp in response to minimal medium was assayed as previously described [7]. The strains were grown over-night in TSB to early stationary phase (12 h), diluted back to an OD_600_ of 0.2 with fresh TSB, and incubated in TSB for additional 2 h, at which point all strains had reached an OD_600_ of 0.3. For nutrient deprivation, 2 ml of each culture were pelleted by centrifugation at 12,000 g for 5 min, and washed once with minimal medium (50 mM MOPS (morpho-linepropanesulfonic acid) pH 7.4, 1 mM MgSO4, 0.25 mM CaCl_2_, 19 mM glutamic acid, and 0.004 mM biotin, 10 mg NAD) and resuspended in 250 μl of minimal medium, ^32^P (Perkin Elmer) was added to 100 μCi/ml, and the culture labeled for 1 h at 37°C. Fifty microliters of labeled culture were added to an equal volume of 2 M formic acid and placed on ice for at least 15 min. The mixture was centrifuged for 5 min at 16,000 g, and 3 μl of the supernatant were spotted directly onto polyethyleneimine (PEI) cellulose thin-layer chromatography plates (Sigma), dried, and developed in 1.5 M KH_2_PO_4_ for 2.5 h. Nucleotides were visualized by autoradiography [24].

### Growth experiments

All strains (S8, S8*ΔrelA* and S8HB) were first grown in 2 ml of TSB for 12 h and diluted with fresh TSB to OD_600_ of 0.2. The diluted cultures were incubated at 37°C. The OD_600_ was determined using an Eppendorf Biophotometer (Eppendorf, Hamburg, Germany) at various time points. The effect of *relA* on the viability of *A*. *pleuropneumoniae* was determined by counting the CFU of *A*. *pleuropneumoniae* at the indicated time points. One hundred microliter aliquots of culture were taken and serially diluted in TSB. After plating in duplicate on TSA plates containing relevant antibiotics and incubated at 37°C for 12 h, the CFU were counted. All experiments were repeated three times.

### Scanning electron microscopy

The pellets of S8, S8*ΔrelA* and S8HB were harvested by centrifugation at 10,000 g after growth in TSB broth for 6 h, 12 h, 24 h and 36 h at 37°C. The harvested cells were washed twice with 0.1 M PBS buffer (pH 7.2) and fixed overnight using 2.5% glutaraldehyde at 4°C. Subsequently, fixed cells were washed three times with 0.1 M PBS (pH 7.2) and dehydrated using increasing concentrations of ethanol (i.e. washed once with each of 50%, 70%, 85%, 95%, and three times with 100%). The samples were subjected to critical point drying with CO_2_ (BAL-TEC CPD030) and metal-spraying (BAL-TEC SCD005) apparatuses. Finally, the cell morphology of all three strains was compared by scanning electron microscope (Hitachi SU8010).

### Quantitative biofilm assay

The microtiter plate biofilm assay is a static assay particularly useful for examining early events in biofilm formation [25]. Overnight cultures were diluted 100× with TSB, and 200 μl of the dilution added to the wells of a sterile 96-well microtiter plate (Costar 3599, Corning, NY, USA). After incubation for 36 h at 37°C, the wells were washed with 200 μl water to remove loosely adherent cells. Excess water was removed by inverting plates several times onto new paper towels. The wells were filled with 100 μl crystal violet (0.1%) and incubated for 2 min at room temperature. After removal of the crystal violet solution, the wells were washed with 200 μl water and dried in a 37°C incubator for 30 min. Subsequently, 100 μl ethanol (70%) was added to each well. Absorbance was measured at 590 nm.

### Confocal laser scanning microscopy

The biofilm assay protocol mentioned above was used except that after washing off non-adherent bacteria, instead of adding crystal violet, 200 μl diluted LIVE/DEAD^@^ BacLight^Tm^ Bacterial Viability Kit solution (Molecular Probes, Eugene, Oregon, USA) were added to stain the bacterial cells. Plates were incubated for 20 min at room temperature in the dark and washed with water. The wells were examined with a confocal microscope (TCS SP5, Leica Microsystems, Hamburg, Germany). SYTO 9 nucleic acid stain was excited at 488 nm and detected using a 520 nm filter. Propidium iodide was excited at 488 nm and detected using a 572 nm filter.

### Physiological and biochemical effects of (p)ppGpp

All strains (S8, S8*ΔrelA* and S8HB) were cultured on TSA plates overnight. Subsequently, bacteria were harvested using PBS, and centrifuged at 12,000 g for 5 min. Pellets were re-suspended to an OD_600_ of 0.2 in the suspension medium provided in the kit. The tests were performed using API identification systems, including API 50 CH, API 20 E, API 20 NE and API ZYM, following the manufacturer instructions (BioMerieux).

### RNA isolation and qRT-PCR

Total RNA was isolated from *A*. *pleuropneumoniae* S8 and S8HB strains grown to early stationary phase (12 h) in TSB broth for analysis of *relA* expression. They were harvested by centrifugation at 10,000 g at 4°C and diluted back to an OD_600_ of 1.0 with fresh TSB medium. The RNeasy kit (Qiagen) was used to isolate RNA. RNA concentrations were measured spectrophotometrically at 260/280 nm (IMPLEN, Germany). Complementary DNA (cDNA) was synthesized using the PrimeScript RT reagent kit with gDNA Eraser (TaKaRa, Japan) following the manufacturer’s instructions. Real-time PCR was performed using a Stratagene3000 system (Agilent Technologies, Germany). The reaction volume was 20 μl, containing 2 μl cDNA template, 10 μl 2μSYBR Green I (TaKaRa) and 0.8 μM of forward and reverse primers. PCR reactions were set up in triplicate. For all amplifications, the cycle conditions were 95°C for 2 min, followed by 40 cycles of 95°C for 15 s and 56°C for 1 min. This experiment was done with three biological replicates, and the average values were taken as the quantitative result. The *gyrA* and *recF* gene were used as internal control. Reaction mixtures lacking RNA were used as negative controls for each set of primers. The primers for amplifying cDNAs of *gyrA*, *recF*, *relA* are presented in Table 1. Relative expression values were calculated as 2^−△(CT target − CT reference)^, where CT is the fractional threshold cycle [22].

### RNA-sequencing analysis

Cultures of S8 and S8*ΔrelA* were grown to early-stationary-phase (12 h) in TSB. The cells were collected at 4°C, the RNeasy kit (Qiagen) was used to isolate RNA, and the Ribo-Zero™ rRNA Removal Kit for Gram-negative bacteria (EPICENTRE Biotechnologies) used to remove rRNA. The remaining RNA was quantified and examined for protein and reagent contamination with a Nanodrop ND-1000 spectrophotometer (NanoDrop, Wilmington, DE, USA). RNA samples showing A_260_/A_280_ ratio of 1.8–2.0, and A_260_/A_230_ ratio above 1.5 were selected for analysis. A total of 20 μg of RNAs for both the S8 and S8*ΔrelA* strains were pooled for cDNA library construction.

Illumina sequencing was performed at the Beijing Genomics Institute (BGI)-Shenzhen in Shenzhen, China (http://www.genomics.cn/index) following the manufacturer’s instructions (Illumina, San Diego, CA). The cDNA libraries were constructed according to Illumina’s protocols and sequenced using the Illumina HiSeq 2000 platform. This experiment was done with three biological replicates.

### Differential expression analysis

The raw sequence reads were filtered using the Illumina pipeline. All of the low-quality reads, reads with adaptor contamination, and reads with only one copy were excluded from the analysis. The clean reads remaining were mapped to the reference sequence of *A*. *pleuropneumoniae* S8 (Genbank accession No. ALYN00000000.1).

To identify the genes affected by deletion of *relA*, the libraries were compared. The number of reads for each coding region was determined, the number of total reads was normalized between the libraries and the ratio of S8 to S8*ΔrelA* reads was calculated. Differentially expressed genes were detected as previously described [26], with a false discovery rate (FDR) threshold of 0.01 [27]. Differences with FDR ≤0.001 and log_2_Ratio absolute value ≥1 were set as the threshold for significant differences in gene expression.

The Blast2GO program was used to obtain GO annotations for molecular functions, biological processes and cellular component ontologies (http://www.geneontology.org). The Kyoto Encyclopedia of Genes and Genomes pathway database (http://www.genome.jp/kegg) was used for pathway assignments. The BlastN program (http://blast.ncbi.nlm.nih.gov/) was used to compare sequences with the *A*. *pleuropneumoniae* serovar 7 strain AP76 reference sequence (Genbank accession No. CP001091.1) for annotation.

### Statistical analysis

Basic statistical analyses were conducted with the SPSS software (SPSS, Inc., Chicago, IL, USA). The Student’s t test was used to determine the significance of the differences in the means between multiple experimental groups. The data were expressed as the mean +/- standard deviation, and values of P<0.05 were considered to be significant.

## Results

### Construction of an *A*. *pleuropneumoniae* S8 *ΔrelA* mutant and its complemented strain

The *relA* insertion mutant of *A*. *pleuropneumoniae* S8, constructed with plasmid pEMOC2*ΔrelA* (Fig 1), was confirmed by PCR and DNA sequencing, and designated as S8*ΔrelA*. Complementation of the S8*ΔrelA* mutant was achieved using the plasmid pLS*relA*, with transformants selected on plates containing chloramphenicol and kanamycin. The complemented mutant was designated S8HB.

### The *ΔrelA* mutant fails to produce (p)ppGpp under nutrient deprivation conditions

**Fig 2:**
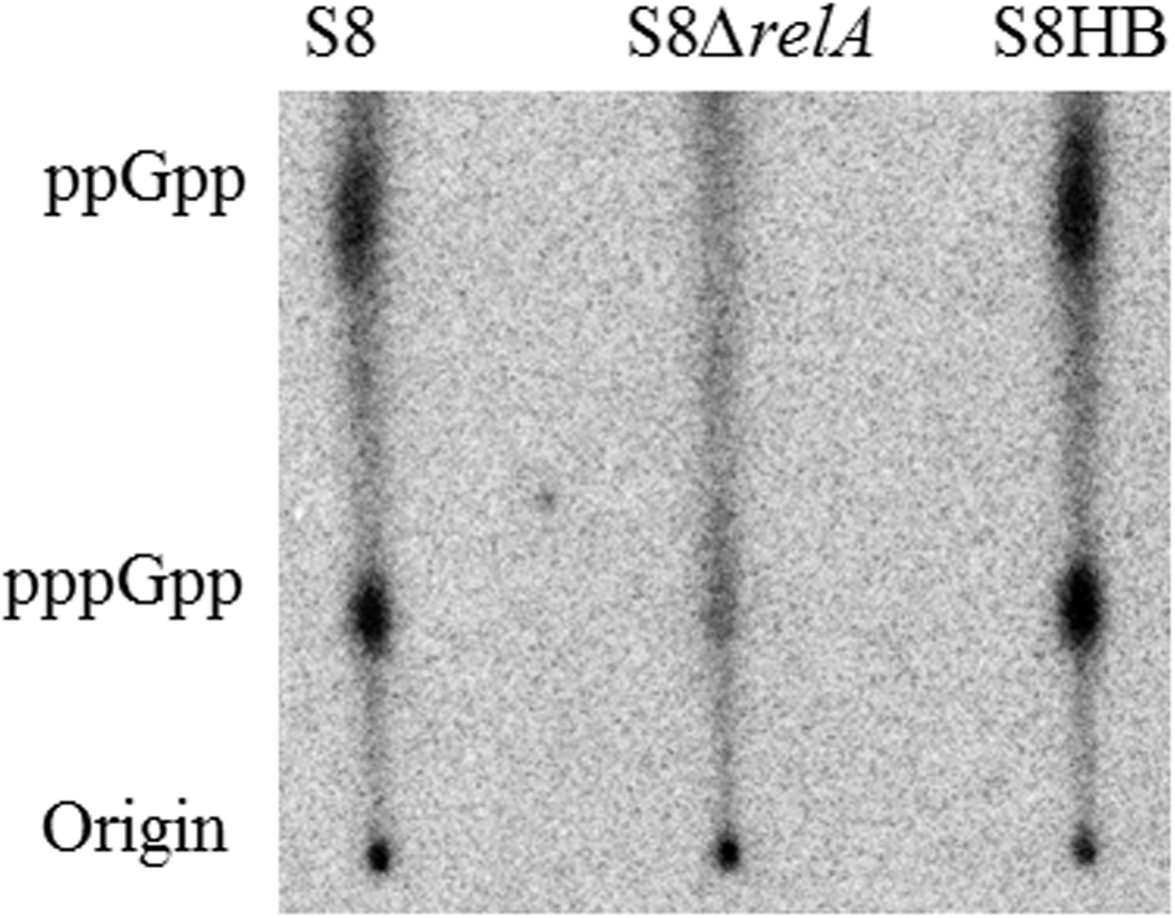
Accumulation of (p)ppGpp in S8, S8*ΔrelA* and S8HB. Cells were labeled with [^32^P]-H_3_PO_4_-labelled in MOPS under starvation conditions, nucleotides were acid extracted, centrifuged, ^32^P_i_-labeled nucleotides were resolved by polyethyleneimine coated TLC plates followed by autoradiography. (P)ppGpp separated by TLC are indicated. Strains used are: S8, S8*ΔrelA* and S8HB.

To determine whether the *ΔrelA* mutant produces (p)ppGpp, all strains were subjected to nutrient deprivation, a condition which had been shown to induce (p)ppGpp [10, 28–30]. S8, S8*ΔrelA* and S8HB were incubated in minimal medium with ^32^P for 1 h. As shown in Fig 2, the S8 and S8HB strains accumulated significant amounts of (p)ppGpp upon exposure to this starvation stress. In contrast, there was almost no detectable level in the S8*ΔrelA* mutant extracts. Plate counts of parallel bacterial cultures after 1 h incubation in minimal medium showed that all three strains remained viable during the experiment; thus, the absence of (p)ppGpp production in the S8*ΔrelA* strain was not due to bacterial death. This result indicated that the *relA* gene product is indispensable for (p)ppGpp production in *A*. *pleuropneumoniae* S8, and disruption of the *relA* gene results in the absence of (p)ppGpp.

### Lack of (p)ppGpp resulted in abnormal growth under nutrient limitation and decreased viability under starvation conditions

**Fig 3:**
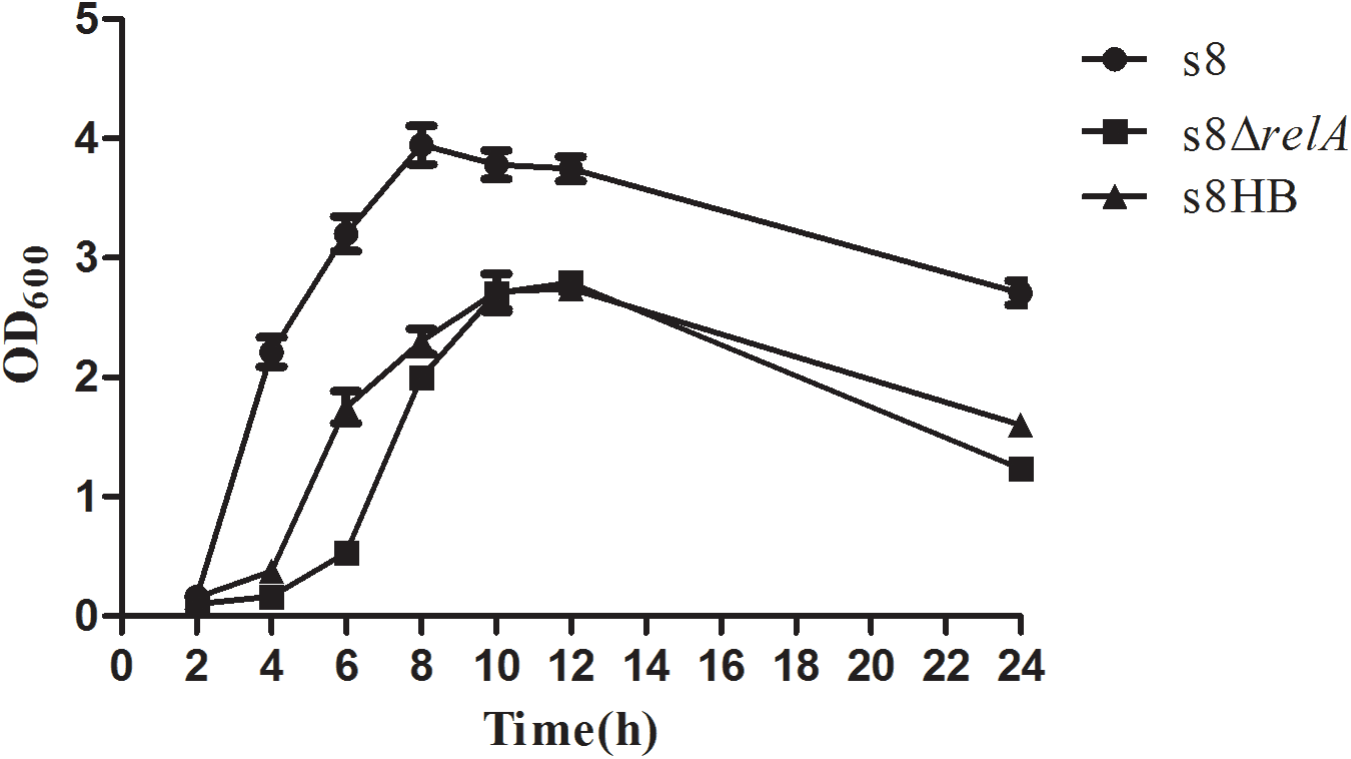
The growth curves of the S-8 strain, S8*ΔrelA* mutant and the complemented S8HB strain. Overnight cultures of S8 (●), S8*ΔrelA* (■) and S8HB (▲)were diluted into fresh medium and then incubated in TSB containing different antibiotics (5 μg/ml chloramphenicol and 50 μg/ml kanamycin). Growth was monitored by OD_600_ at various time points. Points indicate the mean values, and error bars indicate standard deviations.

**Fig 4:**
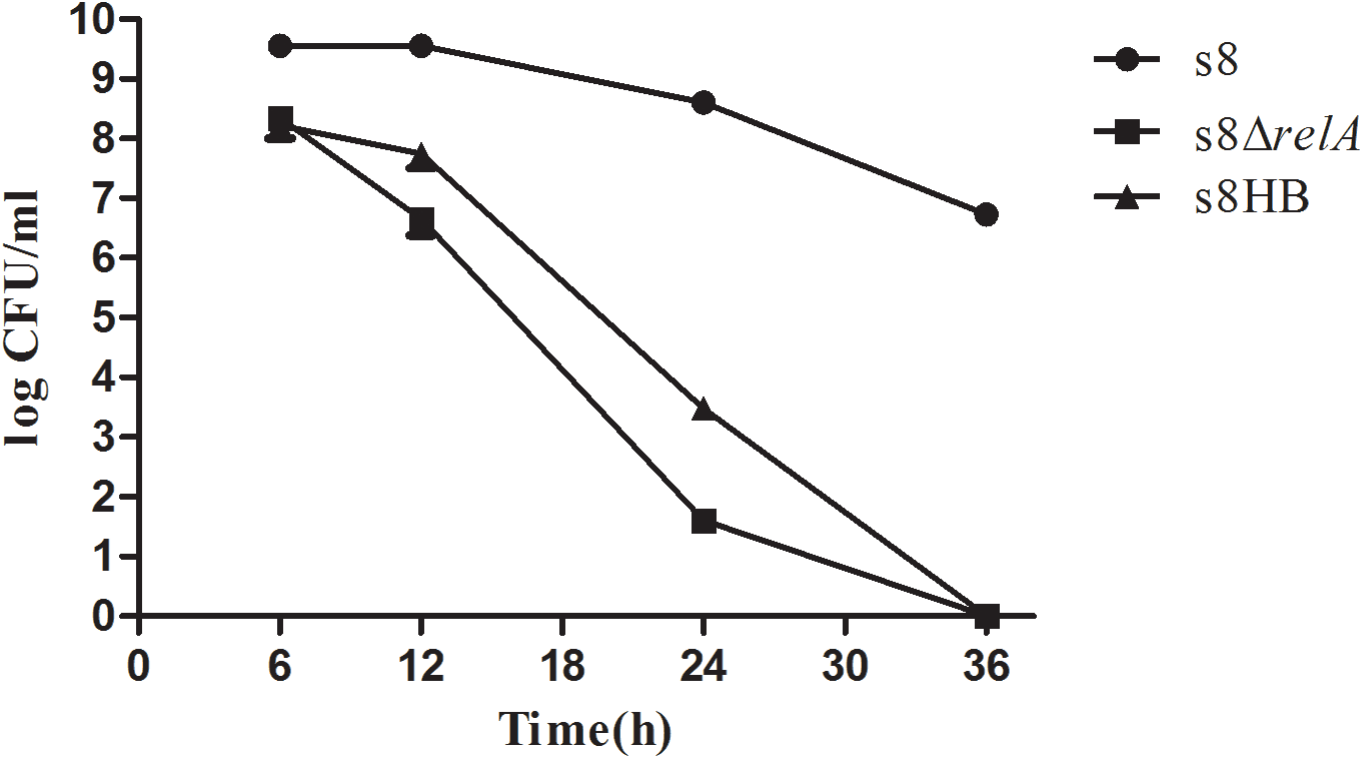
The S8*ΔrelA* mutant has a stationary phase survival defect. S8, S8*ΔrelA* and S8HB strains were grown by shaking under the same conditions at 37°C, and numbers of CFU ml^-1^ were determined at different time points indicated. A representative assay of at least three experiments is shown. Error bars indicate the standard deviations of replicate plating.

Having ascertained that deletion of *relA* affects the production of (p)ppGpp in *A*. *pleuropneumoniae* S8, and given the vital role of the stringent response in bacterial survival, we investigated the impact of (p)ppGpp on cell growth and viability. As shown in Fig 3, OD_600_ measurements indicated that S8*ΔrelA* grew slower than S8 when cultured in TSB. The growth pattern of S8HB was similar to that of S8*ΔrelA*, indicating lack of complementation of the slow-growth phenotype by the cloned *relA* gene. To test whether (p)ppGpp is required for viability, we determined cell counts for S8, S8*ΔrelA* and S8HB cultured in TSB over 36 h. As shown in Fig 4, all strains exhibited a decline in viability from 6 h to 36 h. However, S8 exhibits a gradual decline in viable counts from 12 h to 36 h, whereas, S8*ΔrelA* and S8HB showed a rapid decline during late stationary phase; no viable cells were detected for either strain at 36 h. The results indicated that the *relA* gene expressed on plasmid pLS*relA* was only able to partially restore growth of S8*ΔrelA* in TSB. However, it was unable to complement viability: the number of colonies decreased approximately seven orders of magnitude relative to the S8 strain. These results indicated that (p)ppGpp contributed to prolonged survival under conditions of nutrient limitation.

### Cell morphology of *A*. *pleuropneumoniae* S8 is dependent on (p)ppGpp availability

**Fig 5:**
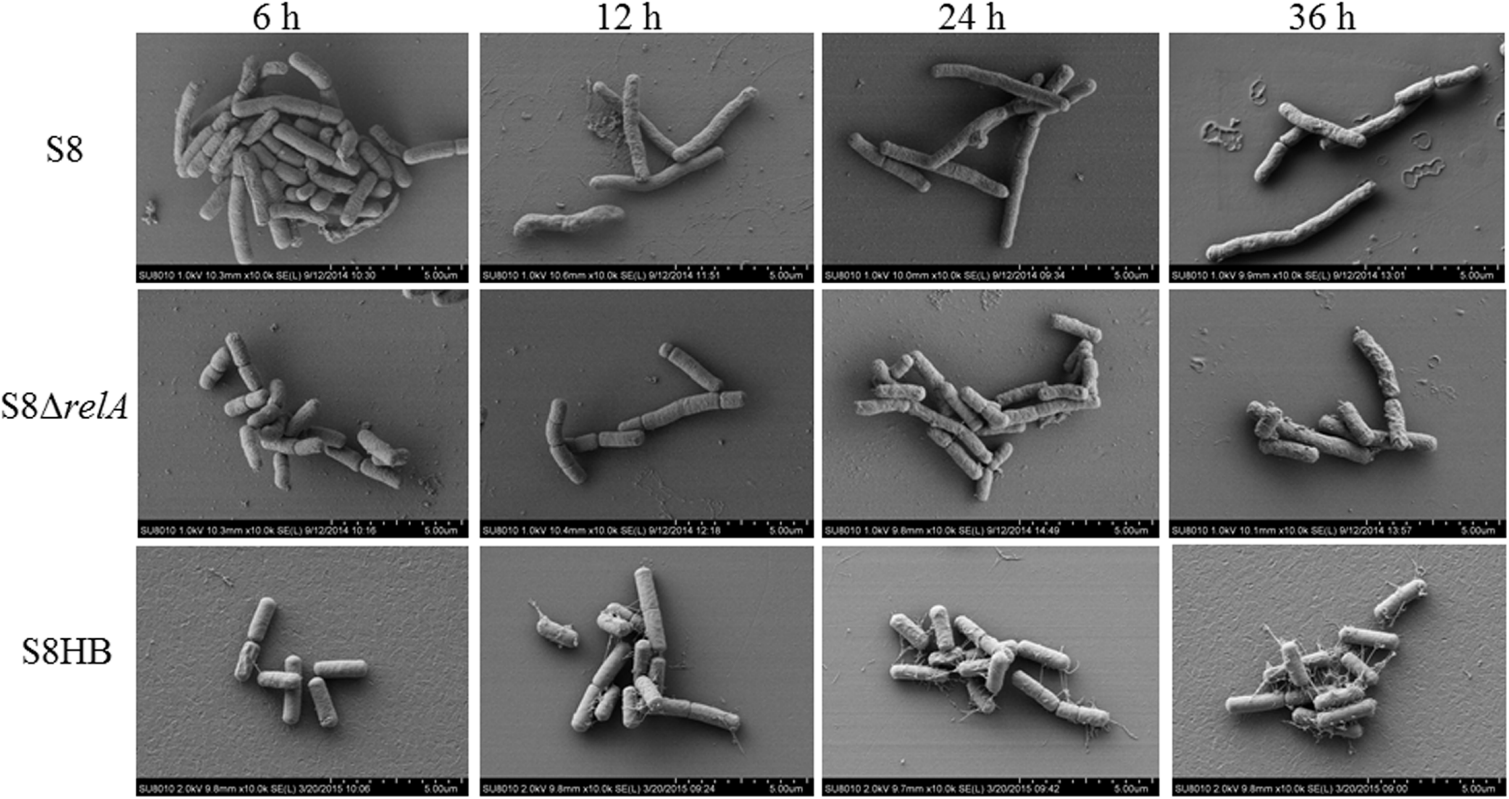
Scanning electron microscopy. SEM of S8, S8*ΔrelA* and S8HB. Compared to the S8, cells of the S8*ΔrelA* mutant and S8HB are of shorter length and typical of WT cells grown in nutrient conditions.

While examining the role of (p)ppGpp in morphology, we found that the S8*ΔrelA* mutant grew as a homogeneous population of non-extended (i.e. short) rods from 12 to 36 h in liquid medium (Fig 5). The non-extended phenotype is in sharp contrast to S8, which grew as a population of significantly longer rods under the same growth conditions. The extended-rod phenotype was not restored by introduction pLS*relA*.

### Deletion of (p)ppGpp synthases affects the biofilm formation of *A*. *pleuropneumoniae* S8

**Fig 6:**
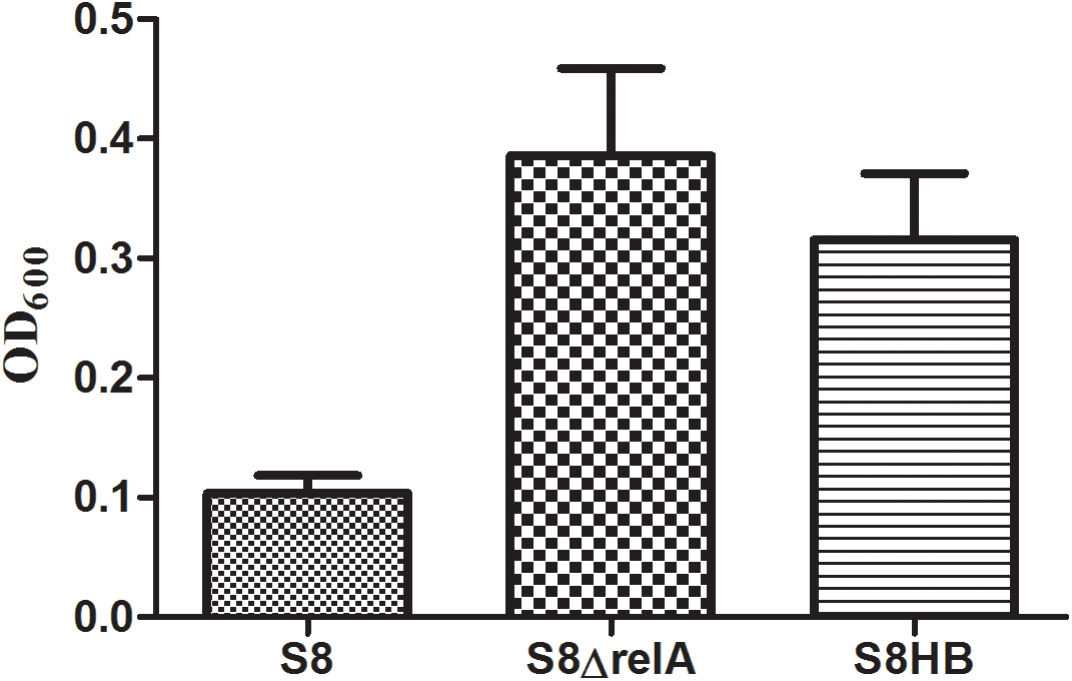
Polystyrene microtiter plate biofilm assay. Biofilm formation of S8, S8*ΔrelA* and S8HB in 96-well polystyrene microtiter plates. The plates were stained with crystal violet. The optical density of the bacterial biofilm formation was monitored by OD_595_ after 36 h incubation. Error bars indicate standard deviations.

**Fig 7:**
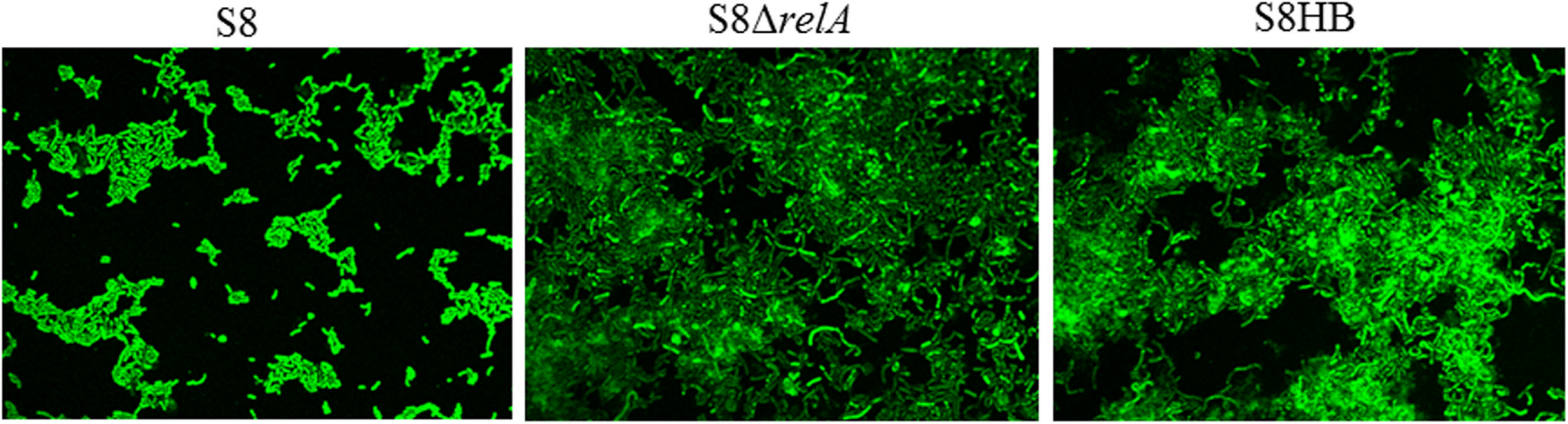
(p)ppGpp affects the biofilm formation of *A*. *pleuropneumoniae* S8. Biofilm development was monitored by confocal laser scanning microscopy after 36 h. The cells were stained with LIVE/DEAD@ BacLightTm Bacterial Viability Kit solution. S8 showed a reduction in biofilm formation compared to the strains S8*ΔrelA* and S8HB.

We tested whether (p)ppGpp plays a role in biofilm formation by examining the biofilm-forming ability of S8, S8*ΔrelA* and S8HB in polystyrene microtiter plates. As shown in Fig 6, S8 and S8*ΔrelA* produced minor and dense biofilms, respectively. Biofilm quantification was confirmed by confocal laser scanning microscopy (Fig 7). These results indicated that (p)ppGpp is involved in *A*. *pleuropneumoniae* biofilm production. The S8HB strain yielded less biofilm than S8*ΔrelA*, although it produced much more biofilm than WT S8 strain (Figs 6 and 7). This indicated that the *relA* gene could not complement the biofilm phenotype when expressed on plasmid pLS*relA*.

### Metabolic effects of (p)ppGpp in *A*. *pleuropneumoniae* S8

To test whether the (p)ppGpp affects the metabolic ability, S8, S8*ΔrelA* and S8HB were cultured in API identification systems. The S8 strain utilizes citrate and all the twelve carbon sources listed in Table 2, and it produces urease. However, S8*ΔrelA* neither uses those carbon sources as substrate, nor produces this enzyme. Introduction of pLS*relA* into S8*ΔrelA* resulted in reversal to wild-type citrate utilization and production of urease, but the production of arginine dihydrolase was not restored (Table 2). No other differences in substrate utilization or enzyme production were seen between S8, S8*ΔrelA* and S8HB.

**Table 2:** Phenotypic characteristic that distinguish the strains of S8, S8*ΔrelA* and S8HB.

Characteristic	S8	S8*ΔrelA*	S8HB
Presence of			
Arginine dihydrolase	+	−	−
Urease	+	−	+
Acid production from			
D-ribose	+	−	+
D-mannose	+	−	+
D-lactose	+	−	+
D-xylose	+	−	+
D-galactose	+	−	+
D-melezitose	+	−	+
D-sucrose	+	−	+
D-raffinose	+	−	+
D-fructose	+	−	+
D-maltose	+	−	+
mannitol	+	−	+
erythritol	+	−	+
Citrate utilization	+	−	+
Gelatin liquefaction	+	−	+

−, negative result; +, positive result. All strains produced acid from D-glucose. None of these strains produced acid from Glycerol, β-methyl-D-xyloside, inositol, α- methyl-D-glucoside, esculin, inulin, glycogen, D-lyxose, α-methyl-D-mannoside, 5-keto-gluconate, L-sorbose, N-acetylglucosamine, salicin, D-melibiose, xylitol, D-tagatose, L-arabinitol, D-arabinose, L-xylose, L-rhamnose, sorbitol, Amygdalin, D-cellobiose, D-gentiobiose, D-fucose, gluconate, L-arabinose, adonitol, dulcitol, D-arabinitol, arbutin, D-trehalose, starch, D-turanose, L-fucose, 2-keto-gluconate. None of these strains had Akaline phosphatase, Leucine arylamidase, Chymotrypsin, N-acetylglucosaminidase, lysine decarboxylase, Acid phosphatase, β-glucuronidase, Ornithine decarboxylase, Lipid esterase(C8), Cystine arylamidase, α-glucosidase, β-fucosidase, Tryptophan deaminase, Lipid enzyme(C14), Trypsase, β-D-glucosidase. All strains had β-galactosidase, Esterase (C4), Valine arylamidase, α-mannosidase, Naphthol-as-bi-phosphate hydrolase, α-galactosidase. All strains were negative by Bile esculin test, H_2_S production, Indole test, VP test. All strains were positive by nitrate reduction and glucose ferment test.

### Validation of expression level of *relA* in S8 and S8HB

**Fig 8:**
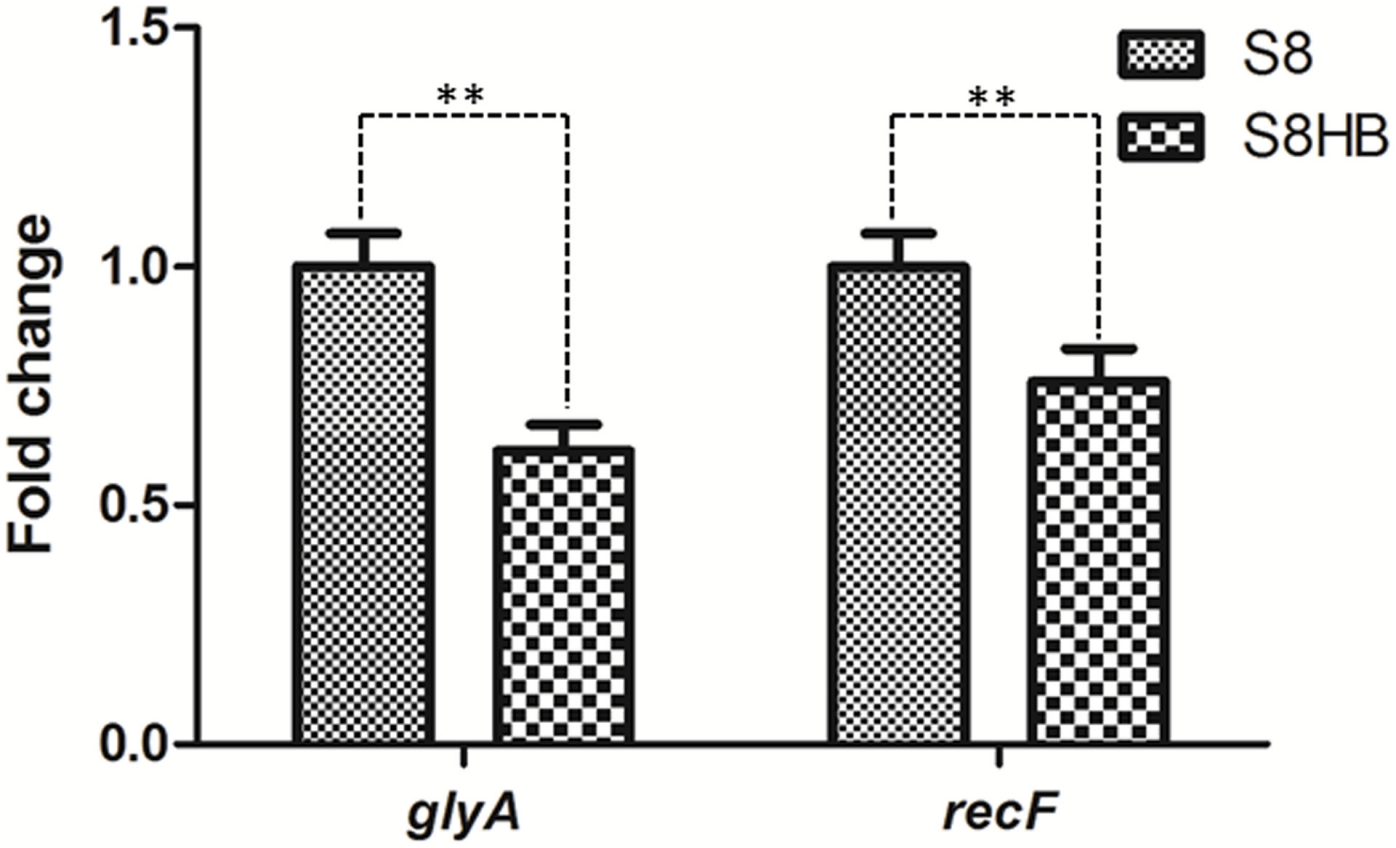
Confirmation of the differentially expressed of *relA* gene by real-time RT-PCR. Transcriptional alteration of *relA* gene was examined by quantitative analysis of corresponding mRNA expression levels in S8 and S8HB. Total RNA were extracted from S8 and S8HB and reverse transcribed into cDNA for subsequent analysis via quantitative PCR. Fold change values were calculated according to the 2^−△△Ct^ method, using *gyrA* and *recF* as internal reference gene. Error bars represent the standard error of three independent experiments, ** represent p-value<0.01.

To compare the expression levels of *relA* in S8 and S8HB, qRT-PCR analysis was performed three times for RNA extracts from the three biological replicates. Two internal reference genes were used to evaluate the expression level. The results indicated that the plasmid encoded *relA* gene had lower expression levels in S8HB than in S8 neither the *glyA* nor *recF* gene as reference gene (Fig 8).

### Global gene expression in the early stationary phase is different in *A*. *pleuropneumoniae* S8 strain compared to the *relA*-deficient mutant

**Fig 9:**
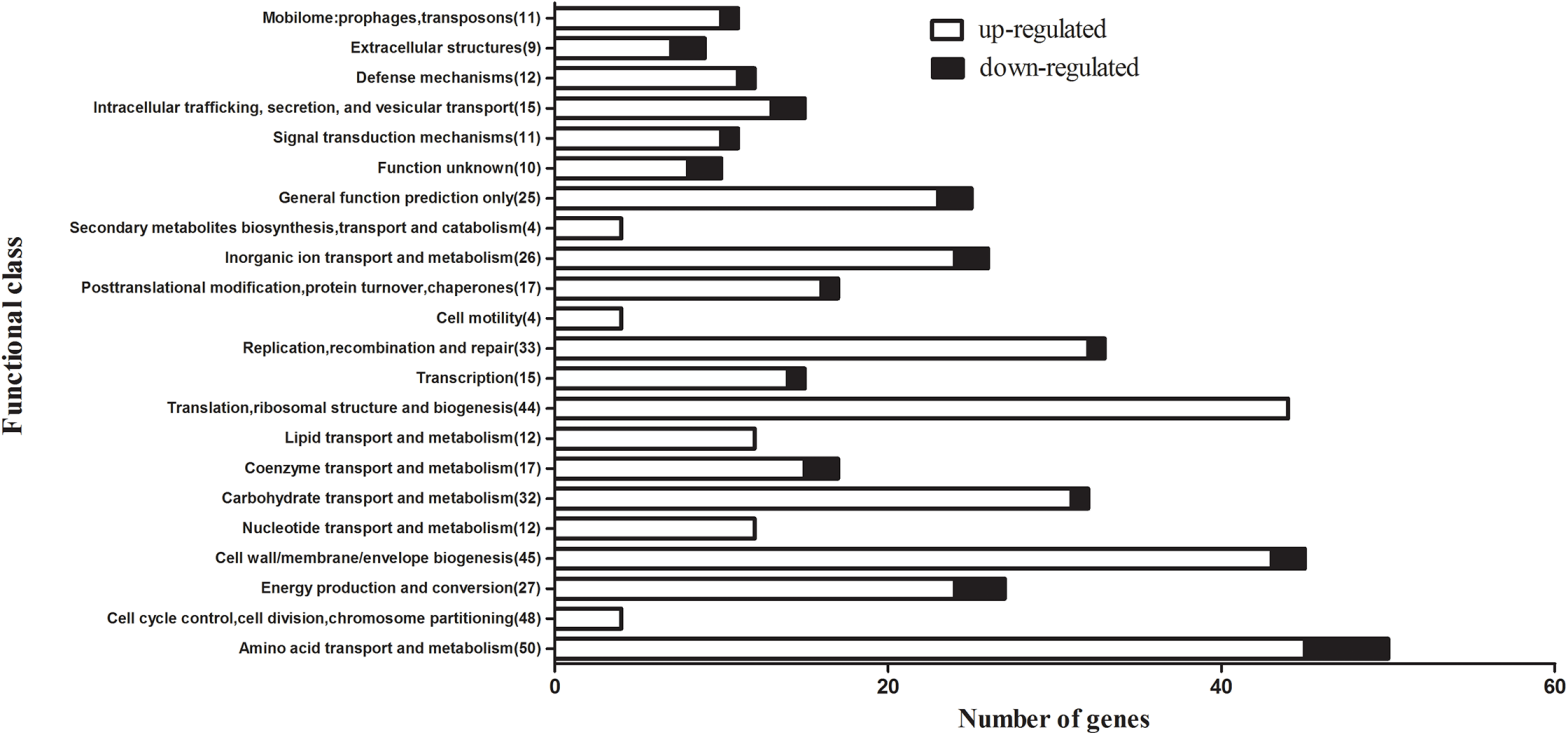
Functional categories of *A*. *pleuropneumoniae* genes that changed their expression profile in the S8 and S8*ΔrelA* during stationary phase growth. Black bars and the white bars indicate the number of genes in each indicated functional category (COG) that was upregulated or downregulated, respectively. The total number of genes within each COG category is indicated in brackets.

To study the effects of the effector molecule (p)ppGpp on a genome-wide scale, the transcriptomes of S8 and S8*ΔrelA* were compared by RNA-seq. The overwhelming majority of the differentially expressed genes in this strain were upregulated (94%). S1 Table shows the differentially expressed genes between S8 and its isogenic S8*ΔrelA* mutant. A total of 405 genes were found to be differentially expressed in the S8*ΔrelA* mutant. Among these, 380 genes were upregulated and 25 genes were downregulated compared to the WT S8. The differently expressed genes were annotated according to the COG database and are summarized in Fig 9. The most prominently upregulated transcription was in genes involved in amino acid transport and metabolism (45/405); cell wall/membrane/envelope biogenesis (45/405); translation, ribosomal structure and biogenesis (45/405); replication, recombination and repair (33/405); carbohydrate transport and metabolism (31/405); energy production and conversion (25/405); inorganic ion transport and metabolism (24/405). The genes coding for a global anaerobic regulator (APP7_0696, log_2_ = 1.04), dimethyl sulfoxide reductase (APP7_1734, log_2_ = 2.38), hemoglobin-binding protein (APP7_1103, log_2_ = 2.26), capsule polysaccharide export protein (APP7_1644, log_2_ = 2.01) and autotransporter serine protease (APP7_0385, log_2_ = 4.03), which are involved in persistence in the upper respiratory tract; the fimbrial biogenesis protein (APP7_0937, log_2_ = 2.96; APP7_0938, log_2_ = 1.38), tight adherence protein (APP7_0589, log_2_ = 1.98), outer membrane protein (APP7_1942, log_2_ = 2.16; APP7_1512, log_2_ = 2.39), which are involved in adhesion to the lower respiratory tract; the ATP-dependent protease (APP7_0400, log_2_ = 4.18; APP7_1329, log_2_ = 2.32), RTX toxin protein (APP7_1051, log_2_ = 1.76), which are involved in the induction of lesions and the ferric hydroxamate receptor (APP7_2103, log_2_ = 1.87; APP7_1452, log_2_ = 1.26), hemoglobin-binding protein A (APP7_1103, log_2_ = 2.26), iron (chelated) ABC transporter periplasmic-binding protein (APP7_0274, log_2_ = 1.56), which are involved in acquisition of essential nutrients, are all upregulated. All of these genes have been characterized as virulence factors in *A*. *pleuropneumoniae* [2, 31–33].

## Discussion

In a previous study by Lone *et al*. [34], genes involved in the stringent response were shown to be up-regulated in a *malT* mutant of *A pleuropneumoniae* grown in porcine bronchoalviolar lavage fluid and it was suggested that that this response may play a role in pathogenesis of this bacterium. However, no further investigations were made regarding the roles of individual genes. In order to investigate the role of RelA, a stringent response regulatory protein responsible for synthesis of (p)ppGpp, in *A*. *pleuropneumoniae* physiology, we constructed a *relA* mutant of the serovar 7 clinical isolate S8 [17]. We confirmed the mutant, S8*ΔrelA*, to be deficient in (p)ppGpp production compared to the WT S8 strain when incubated in minimal medium (Fig 2), and complementation of the mutant by plasmid encoded *relA* (pLS*relA*) was able to restore WT levels of (p)ppGpp under this growth condition.

The S8*ΔrelA* mutant had a reduced growth rate in TSB compared to S8 (Fig 3). This is consistent with previous studies describing *relA*-deficiency leading to growth limitation [35]. However, complementation with the *relA* gene (strain S8HB) only led to partial restoration of growth limitation, possibly due to differences in expression levels of *relA* from the plasmid. Indeed, qRT-PCR analysis of cultures grown in TSB for 12 h revealed that the expression level of *relA* in S8HB was less than in S8 (Fig 8), suggesting lower concentrations of (p)ppGpp in the complemented mutant under these conditions.

It was also found that the viability of S8*ΔrelA* decreased with prolonged culture in TSB compared to S8, i.e it had a stationary phase growth defect. At the 24 h time point, the CFU of S8*ΔrelA* was seven orders of magnitude less compared to S8. No viable S8*ΔrelA* were recovered from the TSB cultures at 36 h when plated on TSA. Other studies have documented that the availability of (p)ppGpp affects bacterial survival, i.e. *relA* deficiency results in the death of the cells. For example, a *Bordetella pertussis relA*-deficient mutant also lost viability more rapidly than the wild-type [36]. In addition, (p)ppGpp is reported to be responsible for significant changes in gene expression, leading to cessation of growth and induction of specific stress responses [12, 37–40]. Our results indicated that (p)ppGpp contributes to prolonged survival of *A*. *pleuropneumoniae* S8 under conditions of nutrient limitation.

In stationary phase in rich medium (TSB), S8 exhibited a change in morphology such that it produced prolonged rods and with an increase in volume (Fig 5), as has previously been seen in other bacteria [41]. Both, S8*ΔrelA* and S8HB had similar morphology to that of non-stationary phase S8 cells (i.e. short rods), even at 36 h in TSB. Transcriptome analysis showed that genes involved in cell wall/membrane/envelope biogenesis were up-regulated in S8*ΔrelA* compared to S8, consistent with the morphological changes observed.

More recently, (p)ppGpp has been reported to be involved in a number of non-stringent processes, including virulence, and biofilm formation [7, 36, 42]. Bacterial biofilm formation is a complex, multifactorial process requiring genes involved in adherence, metabolism, quorum sensing, and stress responses. When we tested the ability of S8, S8*ΔrelA* and S8HB to form biofilms, we found increased biofilm formation by S8*ΔrelA* compared to S8, suggesting that (p)ppGpp negatively regulates biofilm formation of *A*. *pleuropneumoniae* S8. These results are in contrast to those found with *Agrobacterium* [43], *Vibrio cholerae* [7], *E*. *coli* [44], *B*. *pertussis* [36], where (p)ppGpp promoted biofilm formation. The reasons for this apparent discrepenancy are not clear. The transcriptome data revealed that the genes known to be important for the formation of biofilm (*pgaA* log_2_ = 2.73, *pgaB* log_2_ = 1.37, *pgaC* log_2_ = 2.64) were all up-regulated in S8*ΔrelA*. In *A*. *pleuropneumoniae*, biofilm formation has been shown to be part of the extracytoplasmic stress response, with genes of the *pga* operon positively regulated by ϭ^E^ [45]. In addition, ϭ^E^ regulates expression of numerous genes encoding proteins involved in stress response and in reparation and maintenance of the bacterial envelope, and is itself negatively regulated by the anti-sigma factor, RseA [45–47]. However, the *rseA* gene (APP7_0419) was up-regulated (log_2_ = 2.04) in S8*ΔrelA*, suggesting that ϭ^E^ was not the only regulator responsible for biofilm formation in the *relA* mutant.

The stringent response occurs when bacteria encounter nutrient-limited environment and initiates changes in gene regulation in order to maximize the utilization of available resources [30]. In contrast to the growth and morphology results obtained in growth in TSB broth, but in agreement with the detection of WT levels of (p)ppGpp in the S8HB strain when incubated in minimal medium, the metabolic defects (including changes in carbon source utilization and all biochemical characteristics except for arginine dihydrolase; Table 2) in S8*ΔrelA* were complemented in S8HB as determined by API analysis, which uses a minimal medium base for inoculation of the test strips. These results suggest that expression of *relA* from pLS*relA* may be higher in minimal medium than in late log phase in TSB.

Besides the differently expressed genes involved the metabolism, some virulence-related genes were also differently expressed (S1 Table). It has been shown that (p)ppGpp has a direct role in expression of virulence in other pathogenic bacteria [2, 31–33]. Some virulence factors involved in adhesion, acquisition of essential nutrients, induction of lesions, avoiding the host’s defense mechanism and persistence have been up-regulated in S8*ΔrelA*. These results suggest that the virulence of S8*ΔrelA* might be higher than S8, however further experiments are required to validate this.

The gene expression profiles for S8 and S8*ΔrelA* were also analysed to determine if any of the differentially regulated genes could explain the various phenotypes in the *relA* mutant. Some significantly differentially expressed transcripts were detected. Among these, the genes involved in ribosomal structure and biogenesis, amino acid transport and metabolism, and translation cell wall/membrane/envelope biogenesis were all up-regulated in the mutant. This is consistent with a previous report that cells entering stationary phase without (p)ppGpp production have a proteomic profile similar to that during growth [48]. This suggests that the transcriptional program of S8ΔrelA is geared towards exponential growth, and may be poorly adapted to inducing systems that are required to withstand stress. Also, the (p)ppGpp deficient strain failed to suppress unnecessary gene expression under nutrient-limiting conditions, which may contribute to diminished survival. A balanced carbon flux is extremely important for viability of bacterial cells [14].

In conclusion, our results show that during stationary-phase growth in TSB, (p)ppGpp is critical for *A*. *pleuropneumoniae* S8 to adapt to the nutrient-limiting conditions. Several phenotypes were changed owing to the deficiency of (p)ppGpp. By analyzing the transcriptome of a *relA* mutant, we have demonstrated a set of genes that may be regulated by *A*. *pleuropneumoniae* during adaptation to changing nutrient levels. Many differently expressed genes have been identified, and further study will clarify the role of selected genes in the pathogenesis of porcine pleuropneumonia. Some of the genes identified here may serve as new targets in drug and vaccine development.

## Supporting Information

S1 Table The differently expressed genes of S8*ΔrelA* compared to S8 by the deletion of the *relA* gene. All the differently expressed genes were categorized according to the COG database.
(DOCX)
