# Supplementary material for: Role of (p)ppGpp in Viability and Biofilm Formation of Actinobacillus pleuropneumoniae S8
Source: PLoS One. 2015 Oct 28;10(10):e0141501. doi: 10.1371/journal.pone.0141501 (PMC4624843; doi:10.1371/journal.pone.0141501)
Supplement: S1 Table — All the differently expressed genes were categorized according to the COG database. (DOCX) [file pone.0141501.s001.docx]

**S1Table.** **The differently expressed genes of S8*∆relA* compared to S8 by the deletion of the *relA* gene.**

| Gene | Nr-annotation | Fold increase | P-value | COG category |
| --- | --- | --- | --- | --- |
| APP7_1949 | NADH-dependent butanol dehydrogenase | 4.58 | 0 | C |
| APP7_0344 | phosphoenolpyruvate carboxylase | 3.89 | 0 | C |
| APP7_0399 | glycerol kinase | 3.83 | 0 | C |
| APP7_1068 | bifunctional acetaldehyde-CoA/alcohol dehydrogenase | 3.02 | 0 | C |
| APP7_1504 | periplasmic nitrate reductase | 2.57 | 4.39E-18 | C |
| APP7_0730 | trimethylamine-N-oxide reductase precursor | 2.50 | 1.57E-298 | C |
| APP7_1842 | fumarate hydratase class II | 2.42 | 0 | C |
| APP7_1734 | dimethyl sulfoxide reductase | 2.38 | 2.40E-11 | C |
| APP7_1884 | trimethylamine-N-oxide reductase precursor | 2.35 | 0 | C |
| APJL_0601 | NAD(P)H nitroreductase | 2.29 | 1.50E-265 | C |
| APP7_0169 | electron transport complex protein RnfC | 2.29 | 2.74E-103 | C |
| APP7_0859 | phosphoenolpyruvate carboxykinase (ATP) | 1.95 | 0 | C |
| APP7_0403 | anaerobic glycerol-3-phosphate dehydrogenase subunit A | 1.83 | 2.58E-239 | C |
| APP7_0405 | anaerobic glycerol-3-phosphate dehydrogenase subunit C | 1.72 | 1.63E-16 | C |
| APP7_0532 | 2-oxoglutarate dehydrogenase E1 component | 1.56 | 0 | C |
| APP7_1385 | hydrogenase-2 large chain | 1.50 | 6.48E-106 | C |
| APP7_1736 | dimethylsulfoxide reductase, chain B | 1.34 | 5.41E-45 | C |
| APP7_1511 | hypothetical protein | 1.23 | 7.59E-30 | C |
| APP7_0685 | phosphate acetyltransferase | 1.22 | 1.39E-282 | C |
| APP7_0507 | 3-isopropylmalate dehydrogenase | 1.47 | 0 | CE |
| APP7_2126 | glycerate dehydrogenase | 3.34 | 5.57E-18 | CHR |
| APP7_0883 | ABC transporter, CydDC cysteine exporter | 1.45 | 1.91E-05 | CO |
| APP7_0327 | sodium/proton antiporter | 1.39 | 4.05E-218 | CP |
| APP7_0625 | chromosome partition protein MukB | 3.09 | 0 | D |
| APP7_1058 | oligopeptide transporter,periplasmic-binding protein | 1.31 | 5.52E-125 | D |
| APP7_1661 | penicillin binding protein 2 | 2.01 | 0 | DM |
| APJL_0013 | penicillin-binding protein 3 | 1.92 | 5.01E-232 | DM |
| APP7_0547 | tryptophan synthase alpha chain | 4.22 | 0 | E |
| APP7_0186 | T-protein | 3.89 | 0 | E |
| APP7_1370 | PTS system sucrose-specific transporter subunit IIBC | 3.52 | 0 | E |
| APP7_0908 | ABC transporter ATP-binding protein/permease | 2.97 | 0 | E |
| APJL_0869 | FAD-dependent cmnm(5)s(2)U34 oxidoreductase | 2.91 | 1.10E-233 | E |
| Aple02000332 | Uncharacterized kamA family protein | 2.71 | 0 | E |
| APP7_1099 | Aminotransferase AlaT | 2.65 | 2.01E-177 | E |
| APL_1975 | L-2,4-diaminobutyrate decarboxylase | 2.63 | 2.62E-206 | E |
| APJL_0262 | aminoacyl-histidine dipeptidase | 2.54 | 0 | E |
| APP7_0141 | 3-isopropylmalate dehydratase large subunit | 2.32 | 0 | E |
| APP12_900 | Periplasmic dipeptide transport protein | 2.26 | 1.92E-109 | E |
| APP7_0417 | 2-isopropylmalate synthase | 2.23 | 0 | E |
| APP7_1143 | acetylornithine deacetylase | 2.21 | 1.66E-207 | E |
| APP7_2114 | imidazole glycerol phosphate synthase subunit hisF | 2.10 | 1.67E-217 | E |
| APP7_0412 | peptidase B | 2.08 | 1.79E-130 | E |
| APP7_2110 | histidine biosynthesis bifunctional protein hisB | 1.99 | 2.49E-159 | E |
| APP7_1090 | oligopeptidase A | 1.90 | 2.73E-243 | E |
| APP7_2103 | Outer membrane ferric hydroxamate receptor FhuA | 1.87 | 0 | E |
| APP7_0392 | exodeoxyribonuclease V subunit beta | 1.86 | 0 | E |
| APP7_2062 | diaminobutyrate--2-oxoglutarateaminotransferase | 1.81 | 1.30E-242 | E |
| APP7_0005 | Porin Gram-negative type | 1.76 | 3.46E-150 | E |
| APP7_2039 | gamma-glutamyl phosphate reductase | 1.74 | 1.97E-103 | E |
| APP7_0255 | exodeoxyribonuclease V subunit alpha | 1.71 | 0 | E |
| APP7_1956 | MobAB fusion protein | 1.67 | 0 | E |
| APP7_1238 | argininosuccinate lyase | 1.60 | 2.62E-25 | E |
| APP7_0741 | 3-phosphoshikimate 1-carboxyvinyltransferase | 1.53 | 5.79E-140 | E |
| APP7_1556 | integral membrane protein | 1.49 | 7.87E-250 | E |
| APP7_1460 | threonine dehydratase | 1.46 | 0 | E |
| APP7_0334 | 2-octaprenyl-6-methoxyphenol hydroxylase/2-octaprenyl-3-methyl-6-methoxy-1, 4-benzoquinol hydroxylase | 1.38 | 5.13E-128 | E |
| APP7_0271 | threonine synthase | 1.35 | 8.40E-07 | E |
| APP7_2029 | Glutamine synthetase | 1.34 | 1.76E-217 | E |
| APP7_0972 | D-methionine transport ATP-binding protein MetN | 1.30 | 2.11E-41 | E |
| APP7_0215 | Helicase | 1.28 | 2.23E-214 | E |
| APP7_0325 | Methionine synthase II | 1.24 | 2.60E-20 | E |
| APL_0064 | Periplasmic dipeptide transport protein | 1.24 | 6.83E-63 | E |
| APP7_0090 | Xaa-Pro dipeptidase | 2.64 | 0 | EH |
| APP7_0770 | acetolactate synthase large subunit | 1.96 | 3.58E-98 | EH |
| APJL_1897 | ketol-acid reductoisomerase | 1.36 | 0 | EH |
| APL_1656 | Predicted hydrolases of the HAD superfamily | 2.21 | 8.67E-211 | ET |
| APP7_0668 | thymidine kinase | 4.40 | 6.85E-287 | F |
| APP7_0166 | anaerobic ribonucleoside triphosphate reductase | 3.40 | 0 | F |
| APP7_1163 | hypothetical protein | 3.02 | 7.51E-61 | F |
| APP7_1928 | glutamate racemase | 2.97 | 0 | F |
| APP7_0891 | uridine kinase | 2.83 | 4.28E-263 | F |
| APP7_1132 | thiamine biosynthesis protein ThiI | 2.62 | 0 | F |
| APP7_1050 | beta-galactosidase | 2.50 | 0 | F |
| APP7_1045 | Ribonucleoside-diphosphate reductase 1 subunit alpha | 2.17 | 5.05E-24 | F |
| APP7_1011 | IMP cyclohydrolase | 1.47 | 8.07E-68 | F |
| APP7_0750 | exopolyphosphatase | 3.21 | 0 | FTP |
| APP7_1813 | Ser/Thr protein phosphatase family protein | 1.68 | 5.68E-99 | FV |
| APP7_0687 | 2,3-cyclic-nucleotide 2&apos;phosphodiesterase | 1.35 | 7.12E-95 | FV |
| APP7_1195 | glucose-6-phosphate isomerase | 4.59 | 0 | G |
| APL_1565 | Gluconolactonase | 3.79 | 9.28E-227 | G |
| APP7_1039 | transketolase | 3.26 | 0 | G |
| APP7_0692 | phosphomannomutase | 3.20 | 0 | G |
| APP7_2120 | sucrose-6-phosphate hydrolase | 3.18 | 0 | G |
| APP7_1626 | L-xylulose kinase | 3.13 | 2.02E-140 | G |
| APP7_1997 | xylose isomerase | 3.00 | 1.27E-168 | G |
| APP7_1774 | ascorbate-specific permease IIC component UlaA | 2.88 | 0 | G |
| APP7_0062 | transaldolase A | 2.52 | 0 | G |
| APP7_1841 | N-acetylglucosamine-6-phosphate deacetylase | 2.33 | 0 | G |
| APP7_1761 | ascorbate-specific permease IIC component | 2.32 | 0 | G |
| APP7_0636 | phosphoglucomutase/phosphomannomutase | 2.31 | 0 | G |
| APP7_0189 | pyruvate kinase | 2.27 | 0 | G |
| APJL_1873 | sugar phosphate permease | 2.14 | 4.74E-227 | G |
| APP7_0858 | phosphoheptose isomerase | 2.13 | 1.68E-265 | G |
| APP7_1762 | L-ascorbate-6-phosphate lactonase UlaG-like protein | 1.89 | 0 | G |
| APP7_0355 | glycogen phosphorylase | 1.77 | 0 | G |
| APP7_1998 | D-xylose-binding periplasmic protein | 1.58 | 2.24E-51 | G |
| APP7_2014 | triosephosphate isomerase | 1.46 | 8.95E-139 | G |
| APP7_1367 | C4-dicarboxylate transporter | 1.44 | 0 | G |
| APP7_1374 | phosphoenolpyruvate-protein phosphotransferase | 1.38 | 0 | G |
| APP7_1996 | Xylulose kinase (Xylulokinase) | 1.29 | 9.62E-14 | G |
| APL_0343 | Fructose permease IIC component | 1.24 | 3.51E-107 | G |
| APP7_1906 | ribulose-phosphate 3-epimerase | 1.15 | 0 | G |
| APP7_0889 | 2,3-bisphosphoglycerate-dependent phosphoglycerate mutase | 1.08 | 7.29E-73 | G |
| APP7_0850 | Uncharacterized MFS-type transporter | 1.51 | 1.27E-27 | GEPR |
| APL_2003 | Possible outer membrane protein | 2.99 | 0 | GER |
| APP7_1079 | hypothetical protein | 2.60 | 1.34E-12 | GER |
| APP7_2011 | biofilm PGA synthesis lipoprotein PgaB | 1.38 | 1.41E-20 | GM |
| APP7_1545 | pyridoxamine kinase | 2.45 | 6.58E-69 | H |
| APJL_0046 | Riboflavin biosynthesis protein RibF | 2.28 | 4.66E-06 | H |
| APP7_0672 | macrolide-specific ABC-type efflux carrier | 2.16 | 0 | H |
| APP7_0112 | uroporphyrinogen decarboxylase | 2.06 | 0 | H |
| APP7_2057 | Coenzyme A biosynthesis bifunctional protein coaBC | 1.80 | 0 | H |
| APP7_2002 | neutral endopeptidase | 1.55 | 3.54E-248 | H |
| APP7_1138 | S-adenosylmethionine synthetase | 1.25 | 1.98E-26 | H |
| APP7_1525 | glutathione biosynthesis bifunctional protein GshAB | 1.02 | 5.42E-75 | H |
| APP7_0744 | ATP-dependent helicase | 2.32 | 3.81E-212 | HE |
| APP7_0210 | 1-deoxy-D-xylulose-5-phosphate synthase | 2.23 | 0 | HI |
| APP7_1248 | NADPH dehydrogenase | 2.53 | 1.07E-95 | HR |
| APP7_0991 | outer membrane protein | 4.25 | 7.61E-224 | I |
| APP7_0861 | 2-C-methyl-D-erythritol 4-phosphate cytidylyltransferase | 2.92 | 5.08E-298 | I |
| APP7_1251 | 3-hydroxyacid dehydrogenase | 2.66 | 1.20E-203 | I |
| APP7_0959 | lipid A biosynthesis lauroyl acyltransferase | 2.60 | 0 | I |
| APP7_1547 | protein SufI | 2.07 | 0 | I |
| APP7_1164 | glycerol-3-phosphate acyltransferase | 1.36 | 0 | I |
| APP7_1955 | biotin carboxylase | 1.10 | 1.34E-228 | I |
| APP7_1436 | fatty acid/phospholipid synthesis protein PlsX | 1.08 | 5.94E-183 | I |
| APP7_1112 | 3-oxoacyl-(acyl carrier protein) synthase I | 2.55 | 0 | IQ |
| APP7_0421 | long-chain-fatty-acid--CoA ligase | 1.85 | 2.61E-186 | IQ |
| APP7_1916 | prolyl-tRNA synthetase | 3.89 | 0 | J |
| APP7_0614 | ribosome recycling factor | 3.70 | 1.71E-32 | J |
| APP7_2123 | lipoprotein-releasing system transmembrane protein | 3.41 | 0 | J |
| APP7_1889 | glycyl-tRNA synthetase subunit beta | 3.38 | 0 | J |
| APP7_0387 | ATP-dependent RNA helicase | 3.31 | 0 | J |
| APP7_0921 | tRNA pseudouridine synthase C | 3.28 | 7.42E-12 | J |
| APP7_1779 | 50S ribosomal protein L1 | 3.14 | 0 | J |
| APP7_1484 | tyrosyl-tRNA synthetase | 3.08 | 6.57E-173 | J |
| APJL_0884 | Leucyl-tRNA synthetase | 3.01 | 0 | J |
| APL_0866 | hypothetical protein | 2.97 | 0 | J |
| APJL_1179 | Ribosomal large subunit pseudouridine synthase B | 2.88 | 0 | J |
| APP7_1562 | valyl-tRNA synthetase | 2.85 | 0 | J |
| APP7_1899 | macrolide ABC transporter ATP-binding protein/permease | 2.75 | 2.13E-106 | J |
| APP7_0694 | alanyl-tRNA synthetase | 2.74 | 0 | J |
| APP7_1153 | queuosine biosynthesis protein QueC | 2.67 | 2.03E-100 | J |
| APP7_0132 | chelated iron transport system membrane protein YfeC | 2.67 | 0 | J |
| APP7_1234 | histidyl-tRNA synthetase | 2.61 | 0 | J |
| APP7_1977 | protease La-like protein | 2.54 | 0 | J |
| APP7_0317 | 23S rRNA (uracil-5-)-methyltransferase RumA | 2.53 | 4.26E-22 | J |
| APP7_0901 | NADPH-dependent 7-cyano-7-deazaguanine reductase | 2.47 | 8.39E-172 | J |
| APP7_0221 | threonine--tRNA ligase | 2.47 | 2.49E-228 | J |
| APP7_1623 | selenocysteine-specific elongation factor | 2.45 | 0 | J |
| APP7_0044 | isoleucyl-tRNA synthetase | 2.27 | 0 | J |
| APP7_0680 | translation initiation factor IF-2 | 2.26 | 0 | J |
| APP7_0737 | Acetyltransferase | 2.12 | 2.45E-224 | J |
| APP7_0583 | GTP-binding protein LepA | 2.00 | 8.00E-304 | J |
| APP7_0973 | cysteinyl-tRNA synthetase | 1.97 | 7.04E-302 | J |
| APP7_0655 | phenylalanyl-tRNA synthetase beta chain | 1.93 | 0 | J |
| APP7_0721 | soluble lytic murein transglycosylase precursor | 1.84 | 0 | J |
| APP7_1326 | glutamyl-tRNA synthetase | 1.69 | 1.42E-53 | J |
| APP7_1572 | elongation factor Tu | 1.68 | 0 | J |
| APP7_0060 | N6-adenine-specific DNA methylase | 1.68 | 0 | J |
| APP7_1496 | ribonuclease E | 1.67 | 1.96E-66 | J |
| APP7_0961 | tRNA pseudouridine synthase A | 1.61 | 3.76E-48 | J |
| APP7_1848 | 50S ribosomal protein L2 | 1.58 | 0 | J |
| APL_1805 | transposase | 1.56 | 3.71E-11 | J |
| APP7_0715 | asparaginyl-tRNA synthetase | 1.42 | 8.56E-16 | J |
| APP7_0178 | poly(A) polymerase | 1.33 | 7.70E-255 | J |
| APP7_1717 | tRNA uridine 5-carboxymethylaminomethyl modification protein GidA | 1.23 | 2.66E-42 | J |
| APP7_1965 | ribosomal large subunit pseudouridine synthase C | 1.21 | 7.99E-70 | J |
| APP7_1876 | hypothetical protein | 1.20 | 2.72E-103 | J |
| APP7_2020 | outer membrane antigenic lipoprotein B precursor | 1.18 | 0 | J |
| APP7_0870 | hypothetical protein | 1.02 | 4.01E-08 | J |
| APP7_0137 | L-asparaginase periplasmic | 3.14 | 0 | JU |
| APP7_0748 | HTH-type transcriptional regulator | 3.11 | 0 | K |
| APP7_1705 | Uncharacterized protein conserved in bacteria | 3.07 | 0 | K |
| APP7_1148 | transcriptional regulator | 2.23 | 2.59E-06 | K |
| APP13_12670 | LysR family transcriptional regulator | 2.08 | 3.52E-19 | K |
| APP7_0419 | sigma-E factor negative regulatory protein | 2.04 | 0 | K |
| APP7_1483 | RNA polymerase sigma factor rpoD | 2.02 | 8.20E-108 | K |
| APP7_0801 | exoribonuclease 2 | 1.97 | 0 | K |
| APP7_1322 | transcriptional accessory protein | 1.69 | 0 | K |
| APP7_0615 | HTH-type transcriptional regulator | 3.01 | 1.62E-142 | KE |
| APP7_0661 | transcriptional repressor of carbohydrate metabolism | 2.95 | 4.86E-178 | KG |
| APP7_0319 | ATP-dependent helicase HepA | 1.41 | 1.38E-70 | KL |
| APP7_2058 | hypothetical protein | 4.04 | 0 | L |
| APL_0705 | Type III restriction-modification system methylation subunit | 3.52 | 3.14E-54 | L |
| APP7_0463 | recombination-associated protein RdgC | 3.26 | 0 | L |
| APP7_2046 | DNA mismatch repair protein mutL | 2.90 | 0 | L |
| APP7_0467 | phage recombinase | 2.85 | 0 | L |
| APP7_1913 | ATP-dependent DNA helicase recG | 2.76 | 0 | L |
| APP7_0738 | DNA topoisomerase I | 2.76 | 0 | L |
| APP7_0267 | DNA polymerase III subunit gamma/tau | 2.68 | 0 | L |
| APP7_0281 | ATP-dependent DNA helicase | 2.19 | 0 | L |
| APP7_0707 | UvrABC system protein B | 2.15 | 1.45E-164 | L |
| APP7_1482 | DNA primase | 2.00 | 2.00E-304 | L |
| APP7_0384 | uracil-DNA glycosylase | 2.00 | 0 | L |
| APP7_1205 | DNA recombination protein RmuC-like protein | 1.98 | 0 | L |
| APP7_0163 | Recombination-associated protein rdgC | 1.92 | 6.80E-30 | L |
| APP7_0992 | methylated-DNA--protein-cysteineS-methyltransferase | 1.92 | 2.80E-07 | L |
| APP7_0001 | chromosomal replication initiator protein | 1.89 | 4.37E-08 | L |
| APP7_1174 | ATP-dependent DNA helicase RecQ | 1.85 | 0 | L |
| APP7_0550 | DNA polymerase I | 1.69 | 0 | L |
| APP7_0380 | endonuclease 4 | 1.59 | 0 | L |
| APP7_1811 | helicase | 1.58 | 4.17E-44 | L |
| APP7_0146 | ATP-dependent endonuclease | 1.55 | 1.88E-06 | L |
| APP7_0289 | DNA gyrase subunit A | 1.54 | 0 | L |
| APP7_0847 | ATP-dependent helicase | 1.52 | 9.88E-05 | L |
| APP7_0844 | UvrABC system protein A | 1.39 | 3.14E-89 | L |
| APP7_2008 | site-specific recombinase | 1.29 | 9.45E-219 | L |
| APP7_1971 | Exodeoxyribonuclease V, gamma subunit | 1.29 | 0 | L |
| APP7_1353 | DNA ligase | 1.18 | 2.65E-275 | L |
| APP7_1377 | DNA helicase II | 1.12 | 6.81E-47 | L |
| APJL_1357 | Transcription-repair-coupling factor | 1.45 | 0 | LK |
| APP2_0974 | Phage integrase/recombinase | 3.57 | 3.52E-08 | LX |
| APP6_19120 | Integrase | 3.39 | 1.80E-32 | LX |
| APP7_0306 | Cell envelope integrity inner membrane protein TolA | 4.20 | 3.84E-05 | M |
| APP7_1479 | glycosyl transferase family protein | 4.08 | 2.97E-23 | M |
| APP7_1802 | membrane-bound lytic murein transglycosylase C | 3.47 | 3.46E-89 | M |
| Aple02000944 | Exopolysaccharide biosynthesis protein | 3.25 | 3.37E-201 | M |
| APP7_0868 | hypothetical protein | 3.23 | 0 | M |
| APP7_1909 | penicillin-binding protein 1B (PBP1b) | 3.04 | 8.20E-44 | M |
| APP7_0321 | signalling protein AmpD | 2.85 | 2.17E-94 | M |
| APP7_0627 | Possible alkaline phosphatase superfamily hydrolase | 2.70 | 3.34E-229 | M |
| APP7_0793 | MscS family protein | 2.67 | 0 | M |
| APP7_1003 | D-alanyl-D-alanine carboxypeptidase/D-alanyl-D-alanine-endopeptidase | 2.56 | 0 | M |
| APP7_1328 | tetraacyldisaccharide 4&apos;-kinase | 2.54 | 5.42E-248 | M |
| APP7_0435 | protective surface antigen D15 | 2.48 | 0 | M |
| APP7_1512 | outer membrane protein P5 | 2.39 | 4.78E-06 | M |
| APP7_1967 | outer membrane receptor protein, mostly Fe transport | 2.31 | 0 | M |
| APP7_1017 | LPS-assembly protein lptD | 2.28 | 3.18E-289 | M |
| APP7_1942 | Outer membrane protein P5 | 2.16 | 7.38E-05 | M |
| APP7_1475 | O-antigen transporter | 2.11 | 1.88E-64 | M |
| APP7_1190 | 3-deoxy-D-manno-octulosonic-acid transferase | 2.02 | 2.69E-198 | M |
| APP7_1644 | capsule polysaccharide export protein | 2.01 | 6.60E-157 | M |
| APP7_1534 | opacity associated protein A | 1.88 | 0 | M |
| APP7_0792 | MscS family protein | 1.87 | 4.53E-111 | M |
| APP7_1641 | CDP-glycerol:glycerophosphate | 1.78 | 1.45E-74 | M |
| APP7_0019 | UDP-N-acetylmuramate--L-alanine ligase | 1.77 | 0 | M |
| APP7_1693 | adhesin processing HmwC-like protein | 1.73 | 0 | M |
| APP7_0007 | Lipid-A-disaccharide synthase | 1.62 | 4.70E-194 | M |
| APP7_0790 | penicillin-insensitive murein endopeptidase precursor | 1.44 | 5.17E-15 | M |
| APP4_6470 | Outer membrane protein P2-like protein | 1.43 | 0 | M |
| APP7_1084 | hypothetical protein | 1.26 | 7.63E-84 | M |
| APP7_1337 | UDP-N-acetylglucosamine 1- carboxyvinyltransferase | 1.25 | 0 | M |
| APP7_0198 | membrane carboxypeptidase/penicillin-binding protein | 1.23 | 2.86E-90 | M |
| APP7_1982 | prolipoprotein diacylglyceryl transferase | 1.21 | 0 | M |
| APP7_0024 | N-acetylglucosamine deacetylase | 1.17 | 2.28E-82 | M |
| APP7_1509 | inner membrane protein OxaA | 1.08 | 1.51E-26 | M |
| APP7_0016 | UDP-N-acetylmuramoyl-L-alanyl-D-glutamate synthetase | 1.06 | 1.61E-42 | M |
| APP7_0014 | UDP-MurNAc-pentapeptide synthetase | 1.06 | 4.33E-41 | M |
| APP7_0393 | apolipoprotein N-acyltransferase | 1.04 | 3.47E-73 | M |
| APP7_1639 | CDP-glycerol:glycerophosphate | 2.31 | 1.61E-34 | MI |
| APP7_1410 | thiol:disulfide interchange protein DsbD | 1.76 | 0 | MT |
| APP7_0894 | hypothetical protein | 1.92 | 3.51E-48 | MV |
| APP2_1191 | membrane-fusion protein | 1.29 | 6.70E-59 | MV |
| APP7_2012 | biofilm PGA synthesis N-glycosyltransferase PgaC | 2.64 | 7.92E-05 | N |
| APP7_0937 | fimbrial biogenesis protein | 2.96 | 7.12E-23 | NUW |
| APP7_0938 | fimbrial biogenesis protein | 1.38 | 8.03E-05 | NUW |
| APP7_0939 | type 4 prepilin subunit ApfA | 2.70 | 8.01E-15 | NW |
| APP7_1136 | thioredoxin | 4.58 | 0 | O |
| APP7_0400 | ATP-dependent protease La | 4.18 | 0 | O |
| APP7_0698 | stringent starvation protein A-like protein | 3.82 | 0 | O |
| APP7_1069 | 60 kDa chaperonin | 3.44 | 0 | O |
| APP7_1796 | ATP-dependent hsl protease ATP-binding subunit hslU | 3.12 | 0 | O |
| APP7_1255 | esterase | 2.75 | 4.36E-97 | O |
| APP7_0121 | tail-specific protease precursor | 2.65 | 0 | O |
| APP7_1329 | ATP-dependent Clp protease ATP-binding subunit | 2.32 | 6.20E-219 | O |
| APP7_1995 | chaperone protein DnaK | 2.32 | 0 | O |
| APP7_1145 | hypothetical protein | 1.63 | 0 | O |
| APP7_1675 | urease accessory protein UreF | 1.47 | 5.77E-07 | O |
| APP7_1060 | peptidyl-prolyl cis-trans isomerase D | 1.20 | 2.84E-59 | O |
| APP7_0408 | riboflavin biosynthesis protein | 1.19 | 1.99E-299 | O |
| APP7_1381 | carbamoyltransferase HypF | 1.02 | 7.78E-25 | O |
| APP7_0641 | cell division protease FtsH-like protein | 1.02 | 8.33E-113 | O |
| APP7_1629 | transferrin-binding protein 1 Tbp1 | 4.63 | 0 | P |
| APP7_0924 | hypothetical protein | 3.71 | 1.50E-69 | P |
| APP6_0772 | hypothetical protein | 3.35 | 4.72E-146 | P |
| APP13_19970 | Possible alpha/beta superfamily hydrolase | 3.17 | 0 | P |
| APP7_1492 | high-affinity zinc uptake system protein ZnuA precursor | 2.53 | 0 | P |
| APP7_1315 | copper-transporting P-type ATPase | 2.50 | 0 | P |
| APP7_0455 | hypothetical protein | 2.44 | 2.73E-06 | P |
| APP7_1103 | Hemoglobin-binding protein A | 2.26 | 0 | P |
| APP7_1147 | arsenate reductase | 2.20 | 1.38E-05 | P |
| APP7_0969 | transcriptional regulator | 2.12 | 2.65E-34 | P |
| APP7_1220 | Dyp-type peroxidase family | 2.10 | 4.55E-15 | P |
| APP7_0648 | cation-efflux pump FieF | 1.72 | 2.89E-131 | P |
| APP7_1440 | hypothetical protein | 1.70 | 3.49E-13 | P |
| APP7_1408 | K+-dependent Na+/Ca+ exchanger related-protein | 1.66 | 0 | P |
| APP7_1350 | TonB dependent/Ligand-Gated channel | 1.58 | 6.60E-27 | P |
| APP7_0274 | iron (chelated) ABC transporter periplasmic- binding protein | 1.56 | 0 | P |
| APP7_0713 | exodeoxyribonuclease I | 1.53 | 4.58E-46 | P |
| APP7_2068 | Magnesium transporter corA | 1.38 | 3.26E-22 | P |
| APP7_1452 | Ferric transport system permease protein fbpB | 1.26 | 0 | P |
| APL_1259 | phosphate transport system permease protein PstA | 1.26 | 3.98E-25 | P |
| APP7_1608 | hydroxylamine reductase | 1.01 | 1.69E-13 | PC |
| APL_0998 | RTX toxin protein | 2.18 | 1.43E-06 | Q |
| APP7_1051 | RTX toxin protein | 1.76 | 3.29E-24 | Q |
| APP7_0486 | chitinase | 5.03 | 0 | R |
| APP7_0150 | ribonucleoside-diphosphate reductase large chain | 3.49 | 0 | R |
| APP7_0515 | glutamine amidotransferase | 3.45 | 1.14E-124 | R |
| APP7_0709 | iron dependent peroxidase | 3.39 | 0 | R |
| APP7_0772 | antibiotic maturation factor | 2.67 | 0 | R |
| APP7_0069 | hypothetical protein | 2.46 | 2.68E-94 | R |
| APP7_0427 | GTP-binding protein EngA | 2.38 | 0 | R |
| APP7_1553 | Pirin-related protein | 2.20 | 0 | R |
| APP7_0609 | ABC transporter ATP-binding protein | 2.06 | 0 | R |
| APP7_0314 | ABC transporter ATP-binding protein | 2.05 | 0 | R |
| APP7_1636 | hypothetical protein | 1.98 | 1.23E-109 | R |
| APP12_12280 | hypothetical protein | 1.98 | 5.33E-17 | R |
| APP7_0848 | ATPase | 1.98 | 1.84E-26 | R |
| APP7_0965 | hypothetical protein | 1.97 | 0.000188666 | R |
| APP13_6650 | Zn-ribbon-containing, possibly nucleic-acid-binding protein | 1.84 | 9.01E-38 | R |
| APP7_0193 | Na+-dependent transporter | 1.40 | 1.98E-103 | R |
| APP7_2065 | hypothetical protein | 1.14 | 1.29E-37 | R |
| APP7_1792 | hypothetical protein | 1.07 | 1.29E-12 | R |
| APP7_0385 | autotransporter serine protease | 4.03 | 0 | S |
| APP7_0057 | hypothetical protein | 3.36 | 0 | S |
| APP1_2580 | hypothetical protein | 2.85 | 0 | S |
| APP7_1283 | HTH-type transcriptional regulator MalT | 2.71 | 0 | S |
| APP7_0453 | nucleoid-associated protein NdpA | 2.69 | 0 | S |
| APP7_1772 | hypothetical protein | 1.61 | 0 | S |
| APP7_1751 | inner membrane protein | 1.28 | 1.82E-08 | S |
| APP7_1065 | uroporphyrin-III C-methyltransferase | 1.17 | 2.49E-173 | S |
| APP7_0053 | GTP-binding protein | 3.05 | 0 | T |
| APP7_1783 | hypothetical protein | 2.60 | 6.97E-46 | T |
| APP7_1290 | 4-alpha-glucanotransferase | 2.14 | 0 | T |
| APP7_1122 | sensor protein QseC | 2.06 | 1.28E-07 | T |
| APP7_0696 | regulatory protein HlyX | 1.04 | 2.57E-81 | T |
| APL_0405 | GTP pyrophosphokinase | 2.39 | 7.19E-29 | TK |
| APJL_1079 | hypothetical protein | 2.10 | 7.31E-25 | TK |
| APP7_1014 | Large exoprotein involved in heme utilization or adhesion | 13.68 | 3.35E-05 | U |
| APP4_9930 | Hemolysin activation/secretion protein | 4.20 | 3.84E-05 | U |
| APP7_1810 | signal recognition particle protein (sigma-54 like protein) | 2.66 | 0 | U |
| APP7_1125 | preprotein translocase subunit SecD | 1.88 | 4.61E-87 | U |
| APP7_0240 | preprotein translocase subunit SecA | 1.87 | 3.02E-267 | U |
| APP7_0885 | hypothetical protein | 1.48 | 6.32E-252 | U |
| APP7_1398 | cell division protein FtsY | 1.23 | 1.11E-251 | U |
| APP7_0596 | rough colony protein A | 2.72 | 1.95E-183 | UW |
| APP7_0589 | tight adherence protein F | 1.98 | 5.13E-32 | UW |
| APP13_5280 | Autotransporter adhesin | 1.36 | 5.60E-142 | UW |
| APP7_0297 | type I site-specific restriction- modification system, R (restriction) subunit | 3.37 | 0 | V |
| APP7_0295 | type I restriction enzyme EcoR124II specificity protein | 3.32 | 1.82E-19 | V |
| APP7_1531 | type I restriction enzyme EcoEI R protein | 2.84 | 0 | V |
| APP7_0290 | type I restriction-modification system, M subunit | 2.52 | 7.97E-121 | V |
| APL_0838 | ABC transporter-related protein | 2.40 | 3.55E-265 | V |
| APP7_1526 | Type I restriction-modification system, S subunit | 2.24 | 4.38E-17 | V |
| APP7_0216 | CRISPR-associated protein | 1.23 | 9.33E-12 | V |
| APP7_0591 | Flp pilus assembly protein | 2.06 | 2.66E-10 | W |
| APP7_0474 | type I restriction enzyme EcoR124II M protein | 4.25 | 0 | X |
| APP7_0471 | hypothetical protein | 4.22 | 0 | X |
| APP7_0657 | transposase | 2.29 | 8.63E-81 | X |
| APP7_1012 | transposase | 2.28 | 4.06E-16 | X |
| APP7_0806 | primase | 1.69 | 7.98E-43 | X |
| APP13_4940 | hypothetical protein | 1.34 | 1.74E-11 | X |
| APP13_5130 | Predicted phage tail protein | 1.26 | 9.98E-19 | X |
| APP7_0815 | terminase large subunit | 1.25 | 1.03E-05 | X |
| APP7_1963 | hypothetical protein | 4.09 | 0 | 0 |
| APP7_0199 | Tfp pilus assembly protein | 3.75 | 5.69E-42 | 0 |
| APP7_0201 | hypothetical protein | 3.66 | 2.49E-16 | 0 |
| APP2_1482 | two-component response regulator involved in C4-dicarboxylate transport | 3.28 | 7.42E-12 | 0 |
| APP7_0200 | hypothetical protein | 3.25 | 3.96E-20 | 0 |
| APP7_0726 | hypothetical protein | 3.22 | 1.42E-14 | 0 |
| APP7_0181 | hypothetical protein | 3.14 | 2.85E-36 | 0 |
| APP7_0483 | protein ninG | 2.92 | 6.05E-108 | 0 |
| APP2_0476 | hypothetical protein | 2.76 | 1.30E-71 | 0 |
| APP7_2010 | biofilm PGA synthesis protein PgaA | 2.74 | 4.42E-155 | 0 |
| APP13_17620 | hypothetical protein | 2.65 | 7.49E-68 | 0 |
| APJL_0980 | hemolysin activation/secretion protein | 2.57 | 1.06E-09 | 0 |
| APP7_1786 | hypothetical protein | 2.56 | 9.89E-09 | 0 |
| APP7_1495 | hypothetical protein | 2.33 | 2.81E-30 | 0 |
| APP13_7650 | Potential type III restriction enzyme | 2.16 | 5.24E-09 | 0 |
| APP7_0810 | hypothetical protein | 2.05 | 0.00067512 | 0 |
| APP13_16560 | hypothetical protein | 1.99 | 8.59E-23 | 0 |
| APP7_1478 | hypothetical protein | 1.91 | 1.52E-25 | 0 |
| APP7_1477 | Glycosyltransferase | 1.86 | 6.06E-20 | 0 |
| APP7_1767 | hypothetical protein | 1.62 | 7.06E-10 | 0 |
| APP7_1642 | glycerophosphotransferase | 1.61 | 1.49E-26 | 0 |
| APP7_0220 | hypothetical protein | 1.33 | 2.26E-84 | 0 |
| APP4_16590 | Transposase | 1.33 | 2.21E-05 | 0 |
| APP7_0813 | hypothetical protein | 1.13 | 7.19E-77 | 0 |
| Aple02001177 | hypothetical protein | −1.56 | 3.86E-07 | 0 |
| APP7_1919 | Sulfite oxidase and related enzymes | −2.80 | 0 | C |
| APP7_0152 | Na+)-translocating NADH-quinone reductase subunit A | −1.38 | 0 | C |
| APP7_0952 | formate dehydrogenase, nitrate-inducible, major subunit | −1.02 | 6.46E-106 | C |
| APP7_0643 | tyrosine-specific transporter | −2.38 | 0 | E |
| APP7_0106 | bifunctional proline dehydrogenase/pyrroline-5-carboxylate dehydrogenase | −2.26 | 0 | E |
| APP7_1924 | aspartate-ammonia ligase | −2.15 | 0 | E |
| APP7_0568 | transporter | −2.14 | 0 | E |
| APP7_1351 | hypothetical protein | −3.12 | 2.13E-12 | GEPR |
| APP1_5940 | Thiazole biosynthesis protein thiG | −1.62 | 0.000252578 | H |
| APP7_0956 | bifunctional protein FolD | −1.45 | 4.34E-56 | H |
| APP7_1874 | tRNA guanine-N1-methyltransferase | −1.30 | 0 | J |
| APP7_1789 | DNA-directed RNA polymerase subunit beta&apos | −1.36 | 0 | K |
| APP7_0788 | DNA topoisomerase 3 | −2.15 | 0 | L |
| APP13_5080 | Methyl-accepting chemotaxis protein | −1.35 | 0.000110248 | M |
| APP7_0222 | lipoprotein | −1.18 | 0 | M |
| APP7_0598 | flp operon protein B | −3.20 | 0 | OT |
| APP7_1237 | integral membrane sulfate transporter | −1.03 | 7.15E-19 | P |
| APP7_1186 | membrane protein | −1.31 | 2.29E-08 | R |
| APP7_1794 | hypothetical protein | −1.61 | 3.34E-155 | S |
| APP7_1098 | menaquinone-specific isochorismate synthase | −1.50 | 1.14E-45 | S |
| APP7_0104 | autotransporter adhesin | −1.54 | 5.61E-58 | UW |
| APP7_0520 | autotransporter adhesin | −1.45 | 7.62E-115 | UW |
| APP7_1449 | Alpha-hemolysin translocation ATP-binding protein hlyB | −1.57 | 0 | V |
| Aple02001181 | Methyl-accepting chemotaxis protein | −1.71 | 4.54E-05 | X |
